# A disintegrin and metalloproteinase domain 9 facilitates SARS-CoV-2 entry into cells with low ACE2 expression

**DOI:** 10.1128/spectrum.03854-22

**Published:** 2023-09-15

**Authors:** Ivonne Melano, Wei-Chung Cheng, Li-Lan Kuo, Yuag-Meng Liu, Yu Chi Chou, Mien-Chie Hung, Michael M. C. Lai, Yuh-Pyng Sher, Wen-Chi Su

**Affiliations:** 1 Graduate Institute of Biomedical Sciences, China Medical University, Taichung, Taiwan; 2 Ph.D. Program for Cancer Biology and Drug Discovery, China Medical University and Academia Sinica, Taipei, Taiwan; 3 Research Center for Cancer Biology, China Medical University, Taichung, Taiwan; 4 Department of Internal Medicine, College of Medicine, Chung Shan Medical University, Taichung, Taiwan; 5 Division of Infectious Diseases, Changhua Christian Medical Foundation, Changhua Christian Hospital, Changhua, Taiwan; 6 Biomedical Translation Research Center, Academia Sinica, Taipei, Taiwan; 7 Institute of Biochemistry and Molecular Biology, China Medical University, Taichung, Taiwan; 8 Center for Molecular Medicine, China Medical University Hospital, Taichung, Taiwan; 9 Department of Biotechnology, Asia University, Taichung, Taiwan; 10 Institute of Molecular Biology, Academia Sinica, Taipei, Taiwan; 11 International Master’s Program of Biomedical Sciences, China Medical University, Taichung, Taiwan; 12 Department of Medical Research, China Medical University Hospital, Taichung, Taiwan; 13 Drug Development Center, China Medical University, Taichung, Taiwan; National Chung Hsing University, Taichung, Taiwan

**Keywords:** SARS-CoV-2, coronavirus, virus entry, virus-host interactions, infectious disease

## Abstract

Severe acute respiratory syndrome coronavirus 2 (SARS-CoV-2), the etiological agent of the Coronavirus disease-19 (COVID-19) pandemic, utilizes angiotensin-converting enzyme 2 (ACE2) as a receptor for virus infection. However, the expression pattern of ACE2 does not coincide with the tissue tropism of SARS-CoV-2, hinting that other host proteins might be involved in facilitating SARS-CoV-2 entry. To explore potential host factors for SARS-CoV-2 entry, we performed an arrayed shRNA screen in H1650 and HEK293T cells. Here, we identified a disintegrin and a metalloproteinase domain 9 (ADAM9) protein as an important host factor for SARS-CoV-2 entry. Our data showed that silencing ADAM9 reduced virus entry, while its overexpression promoted infection. The knockdown of ADAM9 decreased the infectivity of the variants of concern tested—B.1.1.7 (alpha), B.1.617.2 (delta), and B.1.1.529 (omicron). Furthermore, mechanistic studies indicated that ADAM9 is involved in the binding and endocytosis stages of SARS-CoV-2 entry. Through immunoprecipitation experiments, we demonstrated that ADAM9 binds to the S1 subunit of the SARS-CoV-2 Spike. Additionally, ADAM9 can interact with ACE2, and co-expression of both proteins markedly enhances virus infection. Moreover, the enzymatic activity of ADAM9 facilitates virus entry. Our study reveals an insight into the mechanism of SARS-CoV-2 virus entry and elucidates the role of ADAM9 in virus infection.

**IMPORTANCE:**

COVID-19, an infectious respiratory disease caused by SARS-CoV-2, has greatly impacted global public health and the economy. Extensive vaccination efforts have been launched worldwide over the last couple of years. However, several variants of concern that reduce the efficacy of vaccines have kept emerging. Thereby, further understanding of the mechanism of SARS-CoV-2 entry is indispensable, which will allow the development of an effective antiviral strategy. Here, we identify a disintegrin and metalloproteinase domain 9 (ADAM9) protein as a co-factor of ACE2 important for SARS-CoV-2 entry, even for the variants of concern, and show that ADAM9 interacts with Spike to aid virus entry. This virus-host interaction could be exploited to develop novel therapeutics against COVID-19.

## INTRODUCTION

Initially discovered in late 2019, severe acute respiratory syndrome coronavirus 2 (SARS-CoV-2) is the etiological agent of the coronavirus disease-19 (COVID-19) pandemic, which threatens global public health and the economy. Several vaccines and monoclonal antibodies targeting SARS-CoV-2 Spike protein have currently been authorized in efforts to curb this pandemic. However, the emergence of new variants that harbor mutations in the Spike protein, such as P.1 (gamma), B.1.617.2 (delta), and B.1.1.529 (omicron), has incapacitated the efficacy of the vaccines (1 – 4). Thus, targeting host factors, which are indispensable for virus infection and are more genetically stable, opens an alternative strategy for the development of new antiviral therapies.

Similar to other members of the Coronaviridae family, SARS-CoV-2 relies on host factors for successful infection (5 – 7). To initiate infection of target cells, its Spike protein, composed of the S1 receptor-binding subunit and the S2 membrane-fusion subunit, binds to angiotensin-converting enzyme 2 (ACE2), a primary host cell surface receptor for SARS-CoV-2. Following virus binding, SARS-CoV-2 enters cells via the cell surface or endocytic pathway. In the cell surface pathway, the Spike protein is cleaved by furin at the S1/S2 boundary and transmembrane serine protease 2 (TMPRSS2) at the S2′ site to trigger the fusion of the viral membrane and cellular membranes (8, 9), leading to the direct release of the viral RNA into the cytoplasm (10). In the endocytic pathway, SARS-CoV-2 is internalized into the cytoplasm by endocytosis. Subsequently, the Spike protein is processed by the cysteine peptidase cathepsin L and then fuses with the endosomal membrane to release the viral RNA into the cytoplasm (11, 12).

ACE2 and TMPRSS2 have been recognized to play vital roles in the entry of SARS-CoV-2 (10). However, a recent study surveyed the expression of viral entry-associated genes using single-cell RNA sequencing (scRNA-seq) and found that TMPRSS2 was not expressed in all ACE2+ cells (13). Furthermore, the expression pattern of ACE2 does not match the tissue tropism of SARS-CoV-2 (13, 14). The highest levels of ACE2 expression are found in the small intestine, testis, kidney, heart muscle, and colon (15). Compared to the ileum, ACE2 expression in the lung is lower and relatively limited to type II alveolar cells (13), suggesting that the susceptibility of the lung to SARS-CoV-2 infection may depend on additional and unappreciated cellular factors.

Given that the SARS-CoV-2 Spike protein needs to be proteolytically activated by host cell proteases to achieve successful infection, we performed an arrayed shRNA screen to search for novel proteases important for SARS-CoV-2 entry. Here, we identified a disintegrin and metalloproteinase domain 9 (ADAM9), a member of the ADAM family of type I transmembrane proteins, as a host factor in SARS-CoV-2 Spike-mediated infection. Similar to other ADAMs, ADAM9 is composed of an extracellular region (which contains a prodomain, metalloproteinase, disintegrin, and cysteine-rich domains), a transmembrane sequence, and a cytoplasmic domain. ADAM9 plays an important role in several biological processes, such as cell growth, cell migration, and angiogenesis (16). It has also been identified as an entry factor for the encephalomyocarditis virus (EMCV), which contributes to human myocarditis (17, 18). In this study, we demonstrate that ADAM9 enhances SARS-CoV-2 Spike-mediated entry of the wild type and other variants. Moreover, ADAM9 plays a role in the binding of SARS-CoV-2 pseudotyped particles and coordinates with ACE2 to accelerate SARS-CoV-2 infection. These results reveal a new host factor for virus entry and may provide an alternative strategy for the development of effective antiviral therapies.

## RESULTS

### Identification of ADAM9 as a host factor for SARS-CoV-2 entry

To identify the host proteases involved in viral entry, we first selected human cells that are susceptible to the infection of our SARS-CoV-2 Spike pseudotyped viral particles (Vpp), containing the Spike protein in the envelope and the luciferase gene inside, which can mimic the authentic SARS-CoV-2 viruses during the entry step of viral replication (6). Upon entering the target cells, the luciferase reporter is expressed, and the luciferase activity reflects the number of infected cells. Since SARS-CoV-2 favorably infects the lung and kidney (19, 20), we tested the human embryonic kidney HEK293T cells and human lung adenocarcinoma H1650 and A549 cells by infecting them with mock (“nude” Vpp, as a negative control), SARS-CoV-2 Spike Vpp and VSV-G Vpp (virus particles pseudotyped with the glycoprotein of vesicular stomatitis virus, as a positive control). As expected, the cells were highly susceptible to VSV-G infection. Among the three, only A549 cells were not susceptible to SARS-CoV-2 Spike Vpp infection (Fig. S1A). Therefore, we used HEK293T and H1650 cells for arrayed shRNA screening.

**FIG 1:**
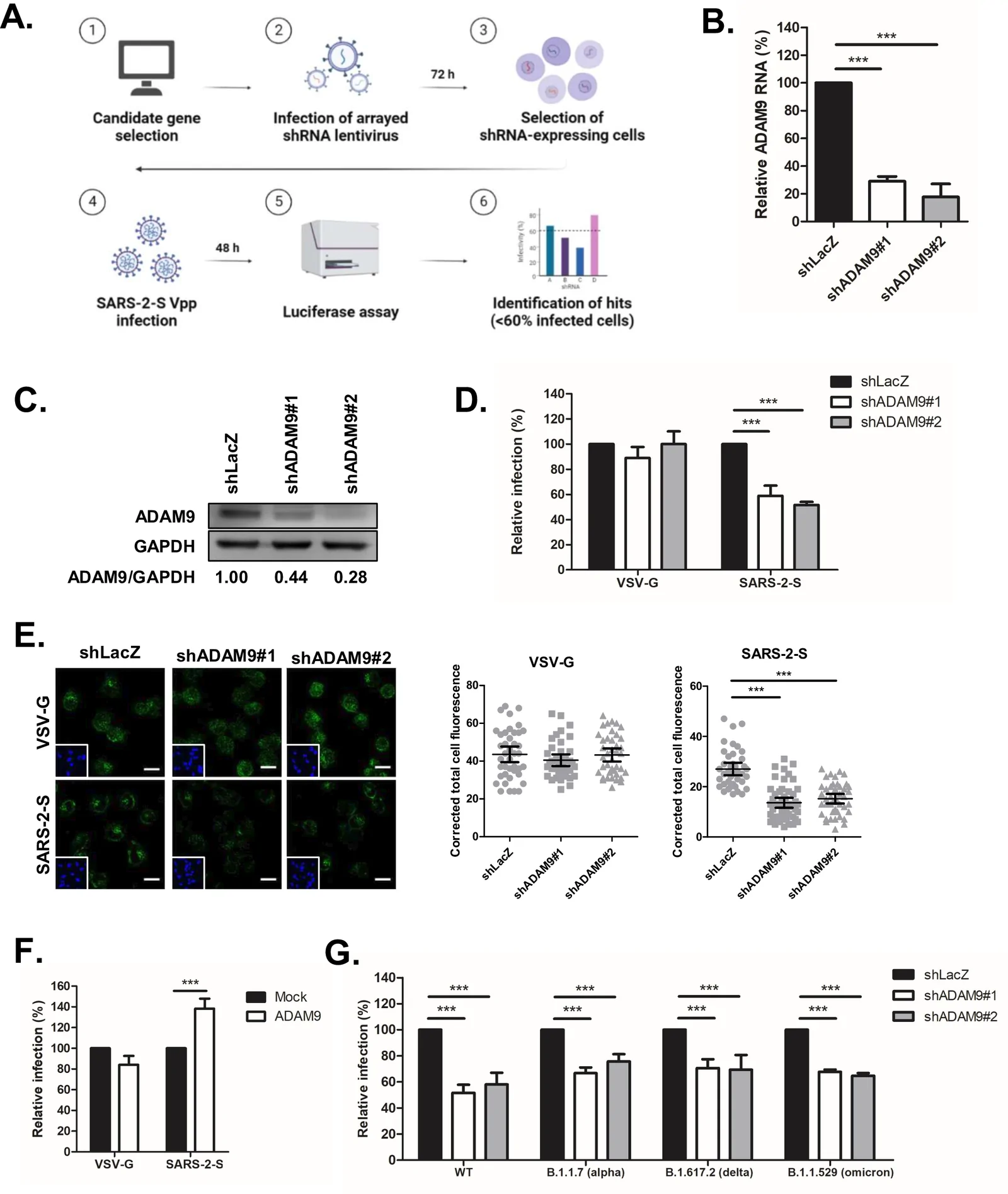
ADAM9 is essential for cellular entry of the SARS-CoV-2 pseudotyped virus. (**A**) Schematic of arrayed shRNA screening procedures in H1650 and HEK293T cells. The figure was created with BioRender.com. (**B and C**) RNA and cellular lysates of shRNA-transduced H1650 cells were extracted after puromycin selection, and ADAM9 expression levels were analyzed by RT-qPCR (**B**) and western blotting (**C**). The level of ADAM9 RNA was normalized by GAPDH RNA. GAPDH was used as the protein loading control. The relative ratio of ADAM9/GAPDH is shown below the blot. (**D**) shLacZ control and ADAM9 knockdown (KD) H1650 cells were transduced with Spike or VSV-G Vpps, and luciferase accumulations were evaluated at 48 h post-infection (hpi). Each infection level was compared with that of the control, shLacZ. (**E**) ADAM9 KD H1650-ACE2 cells were infected with the Vpps at 37°C for 1 h and then fixed for immunofluorescence staining with anti-HIV p24 antibody (green) and DAPI (blue). The corrected total cell fluorescence of at least 40 cells was quantified using ImageJ. (**F**) H1650 cells transfected with ADAM9 were transduced with Spike or VSV-G Vpps at 24 h post-transfection (hpt), and luciferase accumulations were measured at 48 hpi. (**G**) H1650 KD cells were transduced with Spike variant Vpps, and luciferase accumulations were measured at 72 hpi. The values represent the mean ± SD of three independent experiments. *, *P* < 0.05; **, *P* < 0.01; and ***, *P* < 0.001 compared with controls (*n* = 3).

To select host proteins for screening, we used Gene Ontology analysis to choose 860 proteins with endopeptidase-related functions and narrowed them down to 30 candidate proteins with profound localization in the endosome, endolysosome, plasma membrane, membrane raft, or cell surface, since the SARS-CoV-2 Spike protein is processed in the said subcellular locations. We transduced the arrayed shRNA candidates that contained at least two unique shRNAs for each gene into H1650 and HEK293T cells and subsequently infected the shRNA-harboring cells with SARS-CoV-2 Spike Vpps (Fig. 1A). We defined shRNA-targeting genes as “hits” if the Vpp infectivity was reduced to 60% or lower. Notably, the shRNA-targeting genes that have been established as necessary factors of SARS-CoV-2 entry, such as ACE2, cathepsin L (11), and TMPRSS2 (10), were identified, proving the reliability of our method (Table S1, Additional file). Among the top-ranking hits in both H1650 and HEK293T cells, ADAM9, ANXA2, and WNT3A were identified as potential entry factors for SARS-CoV-2. In this study, we chose ADAM9 because (i) it exerts its functions on the cell surface to regulate numerous biological functions (16), (ii) it possesses an enzymatic activity that can be targeted by inhibitors, and (iii) it is involved in the early stages of EMCV infection, another RNA virus (17, 18).

To validate that ADAM9 participates in SARS-CoV-2 entry, we generated stable ADAM9 knockdown (KD) H1650 cells. Two unique shRNA clones targeting ADAM9 reduced ADAM9 RNA to 29% and 18%, respectively, compared to the shLacZ control, which was transduced with an shRNA targeting a non-human gene (Fig. 1B). Correspondingly, the protein expression of ADAM9 was reduced in ADAM9 KD H1650 cells when analyzed by western blotting (Fig. 1C). Upon SARS-CoV-2 Spike Vpp infection, the ADAM9 KD H1650 cells significantly diminished virus infectivity to 59% and 52% (Fig. 1D). We further repeated the knockdown experiments in HEK293T cells. Similarly, the efficient ADAM9 RNA reduction in stable ADAM9 KD HEK293T cells (44% and 34%) markedly decreased SARS-CoV-2 Spike Vpp infection to 46% and 62% (Fig. S1B and C). To further confirm this finding, we knocked down ADAM9 in H1650 ACE2-overexpressing cells (referred to simply as ADAM9 KD H1650-ACE2 cells further in the text), which were subsequently infected with Vpps for 1 h and fixed for immunofluorescence staining against HIV p24 capsid antigen of the Vpps. As shown in Fig. 1E, fewer SARS-CoV-2 Spike Vpp particles were observed in ADAM9 KD H1650-ACE2 cells, indicating that the knockdown of ADAM9 decreases virus entry. The reduction of ADAM9 expression in these cells, but not ACE2, shows that the reduction of virus entry was specifically due to the silencing of ADAM9 (Fig. S1D). These findings are further supported by overexpression of ADAM9 in H1650 cells using HA-tagged ADAM9 plasmid (Fig. S1E), in which transfection of ADAM9 plasmid increased SARS-CoV-2 Spike Vpp infectivity by 38% (Fig. 1F). On the other hand, the infection of VSV-G Vpps was not altered by either the loss or overexpression of ADAM9 (Fig. 1D through F), indicating the specificity of ADAM9 to SARS-CoV-2 Spike entry. One of the challenges of the current vaccines targeting the Spike protein is the frequent emergence of mutations in the Spike protein, thereby limiting the effectiveness of the vaccines. Thus, we investigated whether the infection of SARS-CoV-2 B.1.1.7 (alpha), B.1.617.2 (delta), and B.1.1.529 (omicron) variants would be reduced in ADAM9 KD H1650 cells. As shown in Fig. 1G, a significant reduction in the infectivity of Vpp variants was detected in ADAM9 KD cells. Altogether, these results show that ADAM9 plays a role in SARS-CoV-2 infection, not only for the wild type but also for other variants of concern.

### ADAM9 is involved in the early stages of SARS-CoV-2 entry

**FIG 2:**
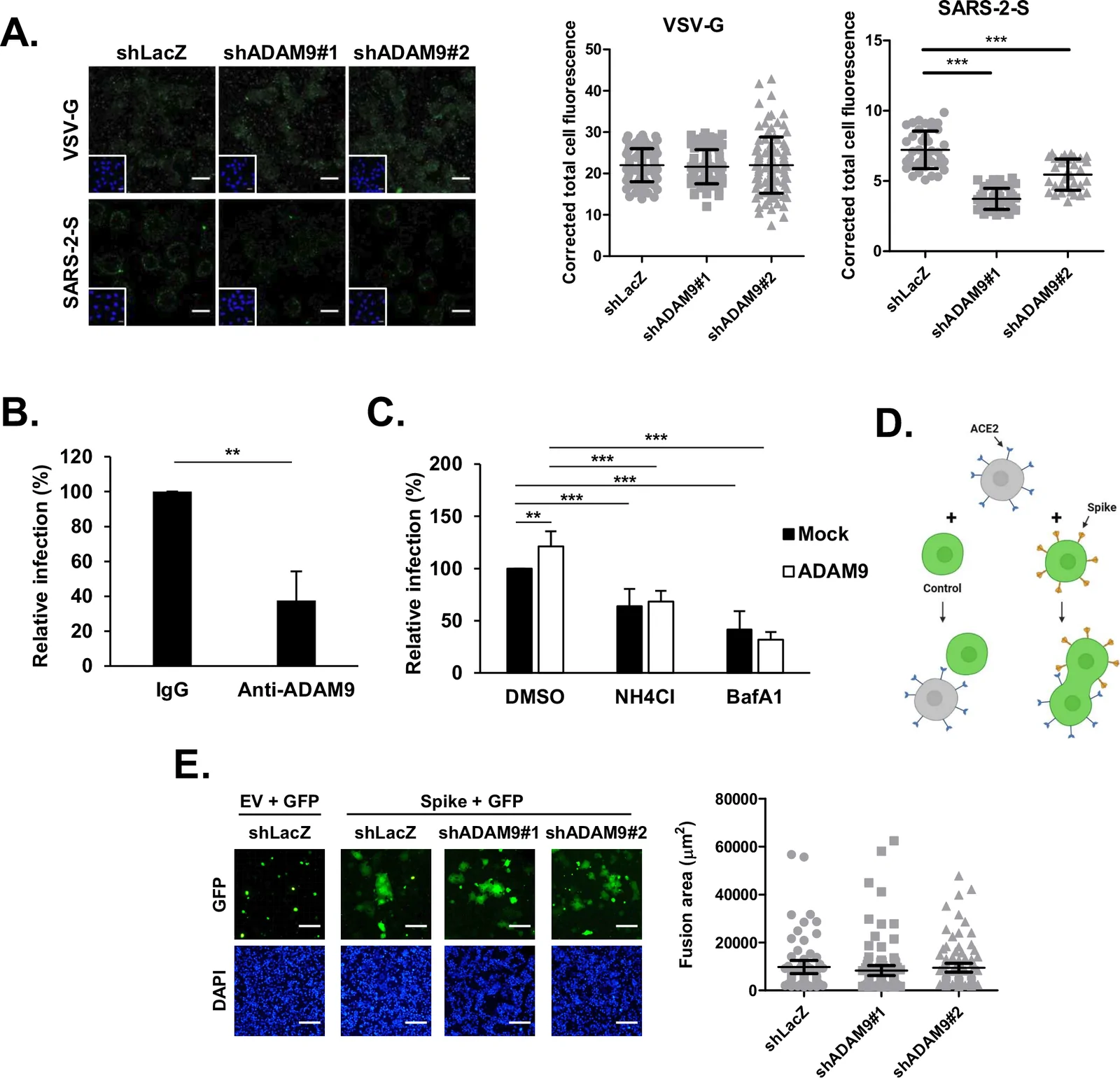
ADAM9 is involved in the early stages of SARS-CoV-2 Spike-mediated entry. (**A**) ADAM9 KD H1650-ACE2 cells were infected with the Vpps at 4°C for 2 h and then fixed for immunofluorescence staining with anti-HIV p24 antibody (green) and DAPI (blue). The corrected total cell fluorescence of at least 33 cells was quantified using ImageJ. (**B**) H1650-ACE2 cells were pretreated with rabbit IgG or anti-ADAM9 antibody for 1 h at 37°C and then infected with Spike Vpps for 1 h at 37°C. Luciferase accumulations were detected at 72 hpi. (**C**) 20 mM NH_4_Cl and 40 nM BafA1 were added to the mock- or ADAM9-transfected H1650 cells for 1 h at 37°C and then infected with the Vpp for 24 h at 37°C. Luciferase accumulations were detected at 72 hpi. (**D**) Illustration of a cell-cell fusion assay. The figure was created with BioRender.com. (**E**) HEK293T cells were co-transfected with Spike and GFP. At 48 h post-transfection, the transfected KD cells were overlaid onto ADAM9 KD H1650-ACE2 cells for 2.5 h at 37°C. The fusion areas of at least 67 syncytia per sample were quantified using ImageJ. Scale bar = 100 µM. The values represent the mean ± SD of three independent experiments. **, *P* < 0.01 and ***, *P* < 0.001 compared with controls (*n* = 3).

To delineate how ADAM9 influences SARS-CoV-2 entry, we explored the effect of ADAM9 at different entry stages, including binding, membrane fusion, and endocytosis. For the binding assay, the stable ADAM9 KD H1650-ACE2 cells were incubated with Vpp at 4°C for 2 h and fixed for immunofluorescence staining to detect bound virions (Fig. 2A). Compared with the shLacZ control, significantly less bound SARS-CoV-2 Spike virions were detected in ADAM9 KD cells, implying that the reduction of ADAM9 decreased the binding of SARS-CoV-2 Spike Vpps, unlike VSV-G, which showed no significant difference in virus binding between shLacZ control and ADAM9 KD cells. To confirm that ADAM9 influences virus attachment, we performed an antibody neutralization assay in H1650-ACE2 cells. As expected, the Spike Vpp infectivity was reduced to 38% when the anti-ADAM9 antibody was used to block cell surface ADAM9 for an hour prior to Vpp infection (Fig. 2B), indicating that ADAM9 facilitates SARS-CoV-2 binding. Next, we sought to investigate whether ADAM9 is also involved in the endocytosis of SARS-CoV-2 Spike Vpp. Given that SARS-CoV-2 can enter cells via the endocytosis pathway, which requires a low pH endosomal environment for viral uncoating, we used two common inhibitors of endosomal acidification, ammonium chloride (NH_4_Cl) and bafilomycin A1 (BafA1), to disrupt the endocytosis pathway (21). In the absence of these inhibitors, ADAM9-transfected cells increased Vpp infection (Fig. 2C). In contrast, pretreatment of NH_4_Cl and BafA1 in ADAM9-transfected cells decreased Vpp infection to comparable levels with mock-transfected cells (Fig. 2C). The abolishment of ADAM9-enhanced Vpp infection by these inhibitors suggests that ADAM9-mediated viral entry requires endosomal acidification. Moreover, it is known that SARS-CoV-2 enters cells by another mechanism, namely, plasma membrane-localized fusion (7). To evaluate whether ADAM9 is involved in this process, we performed a cell-cell fusion assay (6, 22). Briefly, HEK293T cells co-transfected with plasmids encoding individual SARS-CoV-2 Spike and green fluorescent protein (GFP) were co-cultured with ADAM9 KD ACE2-expressing H1650 cells. Once cells harboring GFP and Spike proteins bind to ACE2-expressing cells and fuse, they form a large cell cluster filled with GFP (Fig. 2D). After a 1 h co-culture, large syncytia were formed in ADAM9 KD cells at a comparable level to shLacZ control cells, implying that ADAM9 does not play a role at the fusion stage (Fig. 2E). Altogether, our data suggest that ADAM9 participates in the binding stage of SARS-CoV-2 infection and the ADAM9-mediated entry may be involved in the endocytosis pathway.

### ADAM9 interacts with the Spike S1 subunit

**FIG 3:**
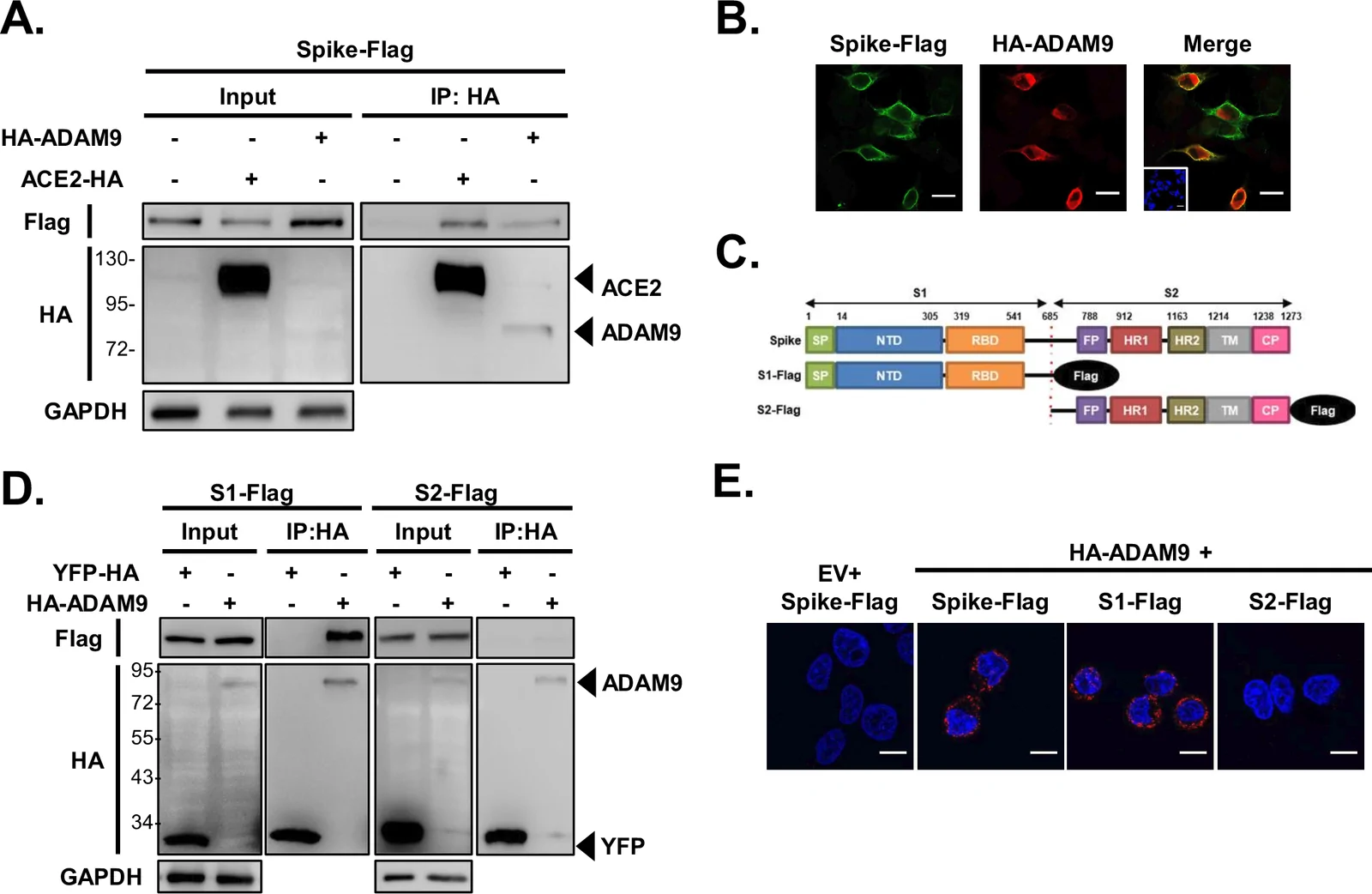
ADAM9 interacts with the S1 subunit of the SARS-CoV-2 Spike protein. (**A**) HEK293T cells were co-transfected with equal amounts of Spike-Flag and pCAG2 mock vector (as a negative control), HA-ADAM9, or ACE2-HA (as a positive control) and harvested at 48 h post-transfection (hpt). Lysates were immunoprecipitated overnight at 4°C using anti-HA agarose and subjected to western blotting using anti-Flag or anti-HA antibodies. (**B**) Equal amounts of Spike-Flag and HA-ADAM9 were co-transfected into H1650 cells. At 48 hpt, the cells were fixed and permeabilized for immunofluorescence staining using anti-Flag (green) and anti-HA (red) antibodies. Scale bar = 25 µM. (**C**) Schematic diagram of SARS-CoV-2 Spike S1-Flag and S2-Flag constructs. SP, signal peptide; NTD, N-terminal domain; RBD, receptor-binding domain; FP, fusion peptide; HR, heptapeptide repeat sequence; TM, transmembrane domain; CP, cytoplasm domain. (**D**) HEK293T cells were co-transfected with either S1-Flag or S2-Flag and with either YFP-HA or HA-ADAM9. The immunoprecipitation procedure was done the same way as mentioned in (**A**). (**E**) HEK293T cells were seeded on poly-L-lysine-coated coverslips and co-transfected with Spike-Flag, S1-Flag, or S2-Flag and equal amounts of empty vector (EV) or HA-ADAM9. At 48 hpt, the cells were fixed for the proximity ligation assay (red fluorescence dots). Scale bar = 10 µM.

SARS-CoV-2 attaches to the target cells by binding its Spike protein to the host receptor on the cell surface. Since ADAM9 mediates Spike Vpp binding to cells (Fig. 2A and B), we next investigated whether ADAM9 interacts with the Spike protein. Here, we co-expressed Flag-tagged Spike (Spike-Flag) protein with HA-tagged ACE2 (ACE2-HA) or HA-tagged ADAM9 (HA-ADAM9) in HEK293T cells and performed immunoprecipitation with anti-HA agarose. As expected, Spike-Flag was co-precipitated with ACE2-HA (Fig. 3A). Notably, Spike-Flag was also co-precipitated with HA-ADAM9, indicating that ADAM9 interacts with Spike protein. Moreover, in Spike-Flag and HA-ADAM9 co-expressing cells, immunofluorescence staining showed that ADAM9 co-localizes with Spike proteins (Fig. 3B). We next examined which Spike subunit associates with ADAM9. We constructed the S1 and S2 domains with Flag tags at their C-termini (Fig. 3C) and co-expressed S1-Flag or S2-Flag protein with HA-tagged yellow fluorescent protein (YFP-HA) or HA-ADAM9 in HEK293T cells. We found that a high level of S1 but not S2 was pulled down with HA-ADAM9, suggesting that ADAM9 interacts with S1 (Fig. 3D). In addition, the red fluorescence signals detected by the proximity ligation assay further demonstrated the specific interaction of ADAM9 and Spike as well as the specific interaction of ADAM9 and S1 (Fig. 3E). These data collectively imply that ADAM9 interacts with SARS-CoV-2 Spike, particularly at its S1 subunit.

### ADAM9 contributes to SARS-CoV-2 infection in ACE2-expressing cells

**FIG 4:**
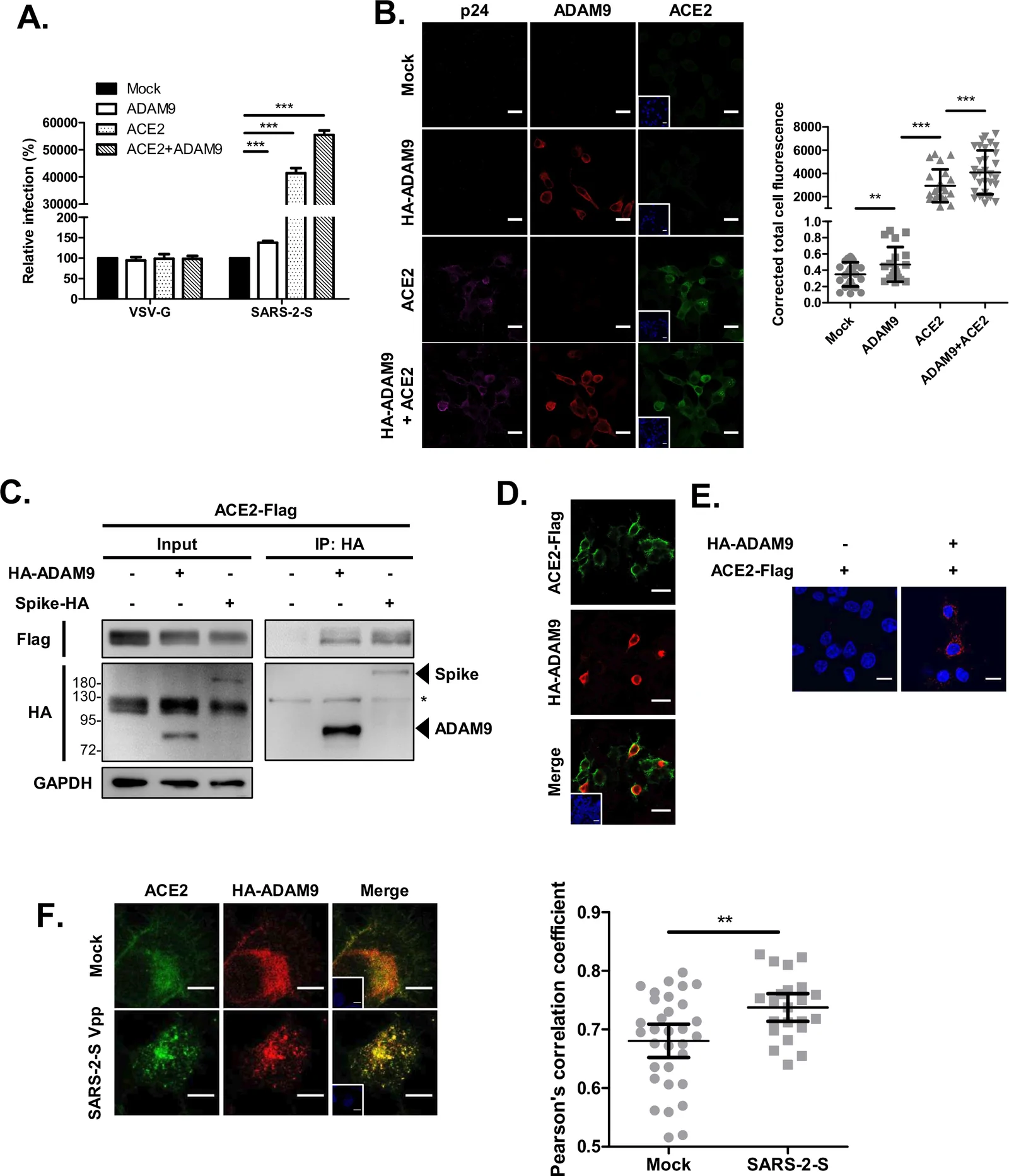
ADAM9 expression enhances SARS-CoV-2 Vpp infection in ACE2-expressing cells. (**A**) H1650 cells transfected with the indicated plasmids were transduced with Spike or VSV-G Vpps at 24 hpt, and luciferase accumulations were measured at 48 hpi. The values represent the mean ± SD of three independent experiments. (**B**) Spike Vpps were bound to the plasmid-transfected H1650 cells for 2 h at 4°C and then fixed for immunofluorescence staining with anti-HIV p24 antibody (magenta) and DAPI (blue). Scale bar = 5 µM. (**C**) HEK293T cells were co-transfected with equal amounts of Flag-tagged ACE2 and pCAG2 mock vectors (as negative controls) and HA-tagged ADAM9 or Spike (as positive controls), and were harvested at 48 hpt. Lysates were immunoprecipitated overnight at 4°C using anti-HA agarose and subjected to western blotting using anti-Flag or anti-HA antibodies. Asterisk: non-specific bands. (**D**) Equal amounts of Flag-tagged ACE2 and HA-tagged ADAM9 were co-transfected into HEK293T cells. At 48 hpt, the cells were fixed and permeabilized for immunofluorescence staining using anti-Flag (green) and anti-HA (red) antibodies. Scale bar = 25 µM. (**E**) HEK293T cells were seeded on poly-L-lysine-coated coverslips and co-transfected with HA-tagged ADAM9 and Flag-tagged ACE2. At 48 hpt, the cells were fixed for the proximity ligation assay (red fluorescence dots). Scale bar = 15 µM. (**F**) H1650-ACE2 cells were transfected with HA-tagged ADAM9. At 24 hpt, the cells were infected with SARS-CoV-2 Vpp for 1 h at 37°C and then fixed for immunofluorescence staining with anti-ACE2 antibody (green), anti-HA antibody (red), and DAPI (blue). Scale bar = 10 µM. (**B**) The corrected total cell fluorescence and (**F**) co-localization were quantified using ImageJ. **, *P* < 0.01 and ***, *P* < 0.001 compared with controls.

ACE2 is widely recognized as the major host receptor for SARS-CoV-2 binding. Since ADAM9 can mediate SARS-CoV-2 binding to H1650 cells, we were curious about the relationship between ADAM9 and ACE2. First, we queried the Genotype-Tissue Expression (GTEx) portal (23) and found that ADAM9 and ACE2 are co-expressed in both the lung and kidney tissues (Fig. S2A). Correspondingly, both of the cells that we used for screening HEK293T and H1650 cells also express both ACE2 and ADAM9 (Fig. S2B). Next, we investigated whether ADAM9 influences the ACE2-mediated SARS-CoV-2 infection by examining the effect of ADAM9 and ACE2 overexpression on Vpp infection in H1650 cells. Similar to Fig. 1F, transfection of ADAM9 alone promoted virus infection up to 138% (Fig. 4A). As expected, overexpression of ACE2 alone boosted Vpp infection (41,378%). Interestingly, a higher Vpp infectivity was detected when ACE2 and ADAM9 were expressed together (55,491%). Similar ACE2 expression in cells transfected with ACE2 alone and with both ACE2 and ADAM9 demonstrated that ADAM9 did not enhance the ACE2 levels for higher Vpp infectivity (Fig. S3A). In contrast, VSV-G infection remains unaffected by the overexpression of the plasmids (Fig. 4A). To explore whether co-expression of ADAM9 and ACE2 enhances virus attachment, a binding assay was performed. As shown in Fig. 4B, more bound virions were detected on cells expressing both ACE2 and ADAM9, compared to ADAM9 or ACE2 alone. These suggest that ADAM9 works alongside ACE2 in promoting SARS-CoV-2 binding. Since ADAM9 and ACE2 are located in the plasma membrane, we next sought to determine whether ADAM9 interacts with ACE2. Flag-tagged ACE2 (ACE2-Flag) and HA-tagged Spike (Spike-HA) or HA-ADAM9 were co-expressed in HEK293T cells and processed for immunoprecipitation assay with anti-HA agarose. Our result shows that ACE2-Flag precipitated with Spike-HA as well as HA-ADAM9 (Fig. 4C), which implies that ACE2 interacts with ADAM9. Immunofluorescence staining also demonstrates that ADAM9 co-localizes with ACE2 (Fig. 4D). Additionally, the proximity ligation assay confirms the interaction between ACE2-Flag and HA-ADAM9 (Fig. 4E). Furthermore, we found enhanced co-localization between ACE2 and ADAM9 upon SARS-CoV-2 Spike Vpp infection (Fig. 4F). Our data collectively suggest that ADAM9 interacts with ACE2 and works alongside ACE2 in enhancing SARS-CoV-2 infection.

### ADAM9 metalloprotease activity is important for SARS-CoV-2 infection

**FIG 5:**
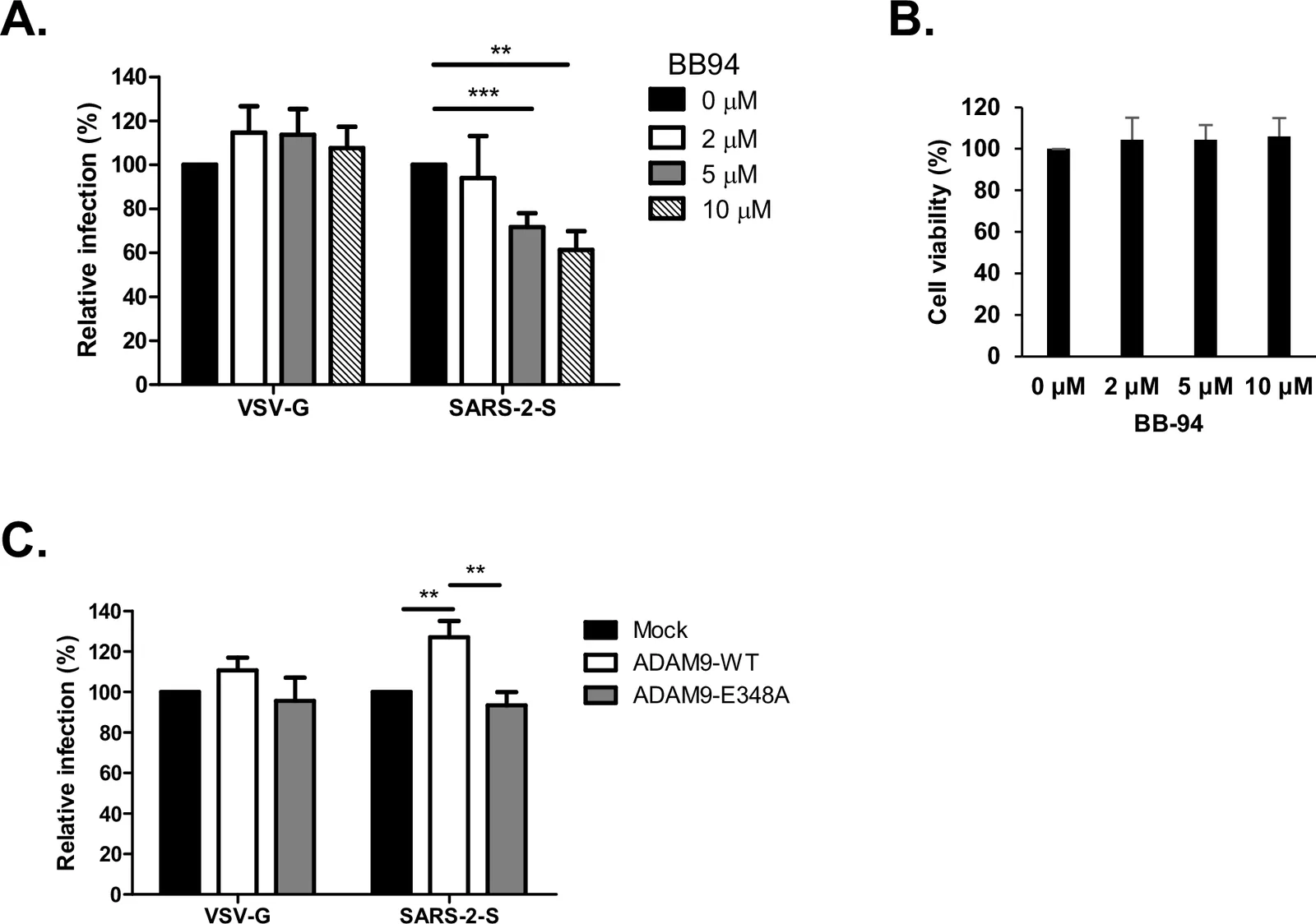
SARS-CoV-2 Spike-mediated entry is dependent on the metalloprotease activity of ADAM9. (**A and B**) H1650 cells were pretreated with BB-94 at the indicated concentrations for 1 h at 37°C and transduced with SARS-CoV-2 Vpp in the presence or absence of the inhibitor in the media. (**A**) Infectivity and (**B**) cell viability were measured using luciferase assay and MTS assay, respectively, at 72 h post-infection (hpi). (**C**) H1650 cells were transfected with HA-tagged ADAM9-WT and ADAM9-E348A. Cells were infected with SARS-CoV-2 Vpp at 24 hpt. Luciferase activity was measured at 48 hpi. The values represent the mean ± SD of three independent experiments. **, *P* < 0.01 and ***, *P* < 0.001 compared with controls (*n* = 3).

To examine whether SARS-CoV-2 Spike-mediated entry requires the metalloprotease activity of ADAM9, we infected the cells in the presence of batimastat (BB-94), a broad-spectrum matrix metalloprotease inhibitor that can block the protease activity of the ADAM family. Entry of SARS-CoV-2 Spike Vpp was efficiently blocked by BB-94 in a dose-dependent manner (Fig. 5A). On the other hand, VSV-G infectivity was not influenced by the presence of the inhibitor. Cell viability assays showed that these concentrations of BB-94 did not induce cytotoxic effects on the cells (Fig. 5B). Furthermore, ADAM9 wild type (ADAM9-WT) significantly increased SARS-CoV-2 Spike Vpp infection but not VSV-G, whereas a catalytically inactive ADAM9-E348A mutant did not affect both SARS-CoV-2 Spike and VSV-G Vpp infection compared with mock (Fig. 5C). The similar protein expression levels of ADAM9-WT and ADAM9-E348A confirmed that the difference in Vpp infectivity is not caused by protein abundance (Fig. S3B). Together, these results indicate that the metalloprotease activity of ADAM9 is important for SARS-CoV-2 infection.

### ADAM9 metalloprotease activity contributes to the endocytosis of SARS-CoV-2

**FIG 6:**
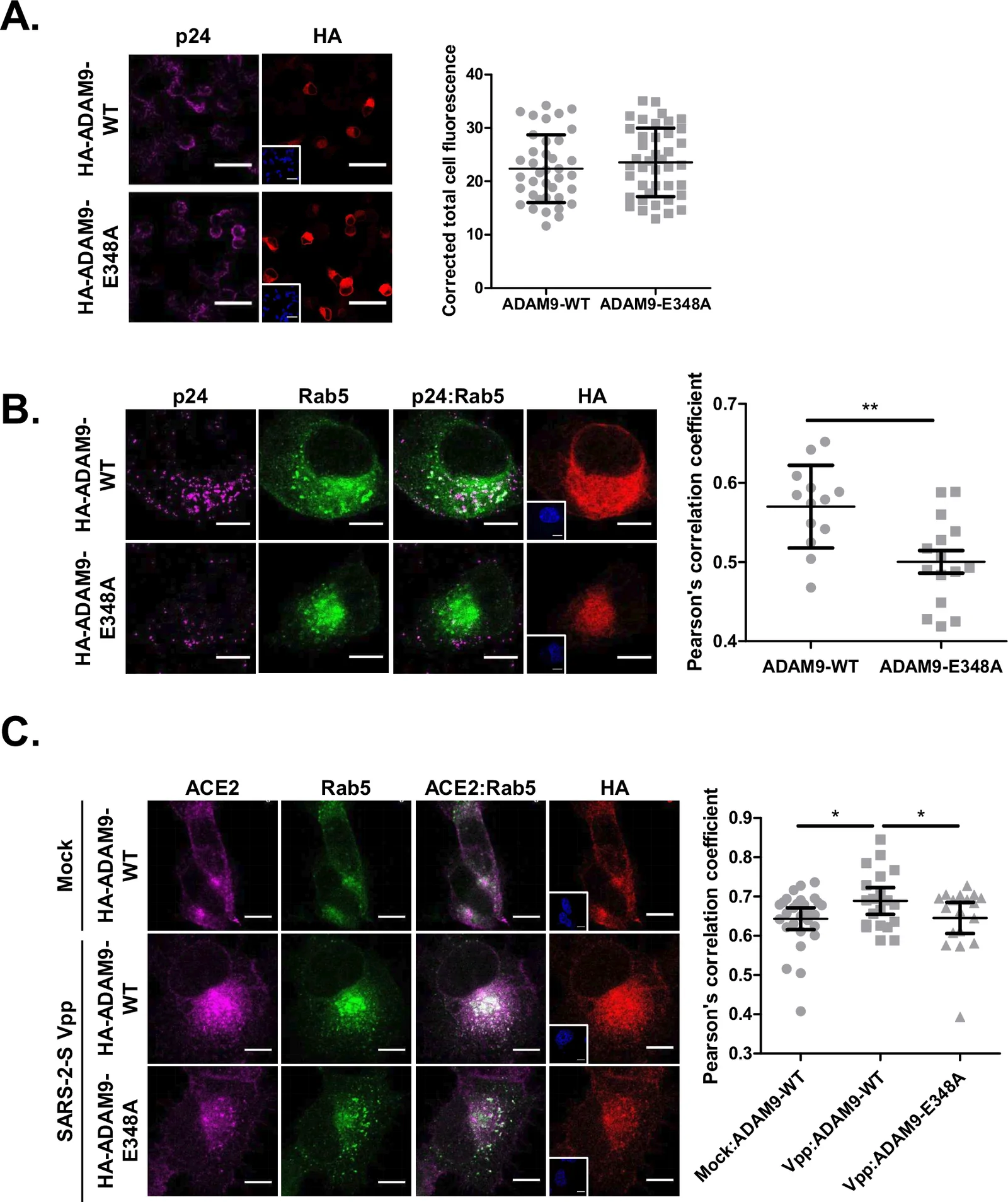
The catalytic activity of ADAM9 plays a role in endocytosis. (**A**) Spike Vpps were bound to the plasmid-transfected H1650-ACE2 cells for 2 h at 4°C and then fixed for immunofluorescence staining against anti-HIV p24 antibody (magenta), anti-HA antibody (red), and DAPI (blue). Scale bar = 50 µM. (**B**) H1650-ACE2 cells were transfected with equal amounts of eGFP-Rab5 and either HA-tagged ADAM9-WT or ADAM9-E348A. At 24 h post-transfection, the cells were infected with SARS-CoV-2 Vpp for 1 h at 37°C and subsequently fixed for immunofluorescence staining against anti-HIV p24 antibody (magenta), anti-HA antibody (red), and DAPI (blue). (**C**) The transfection and infection were done the same way as in (**B**). (**B and C**) Co-localized signals are presented as white points. (**A–C**) Co-localization and corrected total cell fluorescence were analyzed using ImageJ. Scale bar = 25 µM. Results are presented as the mean ± SD.

Since we have demonstrated that ADAM9 functions at the early stages of virus infection (Fig. 2), we next investigated whether the protease activity of ADAM9 participates in SARS-CoV-2 Spike Vpp binding or endocytosis. As shown in Fig. 6A, no significant difference was observed in the amount of bound Vpp virions between ADAM9-WT and ADAM9-E348A-transfected cells, thereby ruling out that the ADAM9 catalytic activity is involved in the binding stage. We further investigated whether the catalytic activity of ADAM9 is crucial in Vpp endocytosis by transfecting the early endosome marker Rab5, which is fused with eGFP (eGFP-Rab5), with either HA-tagged ADAM9-WT or ADAM9-E348A into H1650-ACE2 cells. Immunofluorescence staining revealed significantly less co-localization between SARS-CoV-2 Spike Vpp and Rab5 in ADAM9-E348A-transfected cells than in ADAM9-WT-expressing cells (Fig. 6B). This implies that catalytic activity is important in virus endocytosis. SARS-CoV-2 Spike is endocytosed together with ACE2; hence, we further examined whether ADAM9 catalytic activity influences ACE2 trafficking to the endosomes. Upon virus infection, the ADAM9-WT-transfected cells showed significant enhancement of co-localization between ACE2 and Rab5 compared to mock-transfected cells, proving that ACE2 was indeed endocytosed after virus infection (Fig. 6C). On the contrary, this enhancement was abolished in ADAM9-E348A-transfected cells, suggesting that the catalytic activity of ADAM9 contributes to ACE2 endocytosis. Altogether, these data infer that the metalloprotease activity of ADAM9 influences SARS-CoV-2 and ACE2 trafficking.

## DISCUSSION

Identification of host factors involved in virus entry is valuable not only for gaining mechanistic insights into the virus cycle but also for the development of novel antiviral strategies. RNAi screening is a powerful approach to search for unknown factors that are involved in the virus life cycle (24 – 31). In the current study, we identified ADAM9 as a candidate host protease that is important for SARS-CoV-2 infection by performing an arrayed shRNA screen in H1650 and HEK293T cells, which have low ACE2 expression (32). Moreover, we revealed that ADAM9 binds to the S1 subunit of SARS-CoV-2 Spike protein and functions alongside ACE2 to facilitate virus attachment to the host cell.

Several risk factors, such as hypertension, diabetes, and obesity, have been associated with COVID-19 severity (33). Recently, ADAM9 was identified as a key driver of SARS-CoV-2 severity using the multi-omics analysis of a young, comorbidity-free COVID-19 patient cohort to identify host genes that drive disease severity (34). A higher ADAM9 RNA expression level was detected in the serum of critically ill COVID-19 patients (34). They detected markedly lower intracellular SARS-CoV-2 virus when they silenced ADAM9 in Vero 76 and A549-ACE2 cells, consistent with our findings of the role of ADAM9 in enhancing SARS-CoV-2 virus infection. Besides, they showed higher levels of the soluble form of the major histocompatibility complex (MHC) class I-related chain A (MICA) protein, an ADAM9 substrate (35), in critically ill patients, suggesting that ADAM9 protease activity was high. Consistently, we found that the protease activity of ADAM9 was involved in SARS-CoV-2 virus infection. Moreover, we provided a molecular mechanism linking ADAM9 and SARS-CoV-2 viral infection and the effect of silencing ADAM9 on SARS-CoV-2 variants.

Coronaviruses are prone to encountering mutations in their genome during viral replication (36). Hence, numerous SARS-CoV-2 variants have emerged since the beginning of the pandemic. Currently, the World Health Organization has designated five variants as variants of concern—alpha, beta, delta, gamma, and omicron—because they possess increased transmissibility, virulence, or immune evasion properties. Silencing ADAM9 had varying inhibitory effects on different variants, potentially due to mutations in their Spike proteins, which may affect the ADAM9-Spike interaction. Nonetheless, significant inhibition of Vpp infection by ADAM9 knockdown was observed across the alpha, delta, and omicron variants. Therefore, targeting ADAM9 poses a promising strategy to protect the host from SARS-CoV-2 infection, irrespective of the current Spike mutations.

Even though ACE2 is the major SARS-CoV-2 receptor, several host proteins have been recently discovered as potential alternative receptors or auxiliary attachment receptors for SARS-CoV-2 entry (37). For instance, Spike protein employs AXL or CD147 in ACE2-deficient cells to enter cells via endocytosis (38, 39). The furin-cleaved C-terminal motif in S1 can also bind to neuropilin 1, and the inhibition of this interaction reduces SARS-CoV-2 infectivity (40, 41). In addition, SARS-CoV-2 RBD can bind to kidney injury molecule-1 (KIM-1) and glucose-regulatory protein 78 (GRP78), with the latter regulating ACE2 surface expression as well (42, 43). Moreover, the expressions of asialoglycoprotein receptor 1 (ASGR1) and kringle-containing transmembrane protein-1 (KREMEN1) correlate with SARS-CoV-2 tropism, and both proteins bind efficiently to Spike domains (44). In this study, we propose a possible mechanism for ADAM9’s involvement in SARS-CoV-2 viral entry. Given that the manipulation of ADAM9 expression only had a modest effect on Vpp entry as compared to ACE2, we propose that ADAM9 functions as a co-receptor or an auxiliary attachment receptor of SARS-CoV-2. Likewise, we found that the infectivity of SARS-CoV-2 Vpp in H1650 and HEK293T cells was in proportion to the RNA expression levels of ACE2, but not ADAM9, indicating that virus entry in these low ACE2-expressing cells is primarily dependent on ACE2, while ADAM9 acts as a contributing factor. Upon virus binding to cells, SARS-CoV-2 utilizes its Spike to interact with ACE2, which may be enhanced by ADAM9 interacting with Spike. Additionally, because ADAM9-mediated SARS-CoV-2 infection could be inhibited by NH_4_Cl and BafA1 and the metalloprotease activity of ADAM9 is involved in the endocytosis of SARS-CoV-2 Vpp and ACE2, the interaction of ADAM9 with Spike and/or ACE2 proteins may prompt the endocytosis process, which is supported by studies that demonstrate ADAM9 binding could mediate β1 integrin endocytosis and its metalloprotease activity may be involved in the growth factor-dependent endocytosis of E-cadherin (45, 46). ADAM9 can be internalized via clathrin-mediated endocytosis (47); however, how ADAM9 mediates the endocytosis of surface proteins remains unclear. On the other hand, we could not rule out the possibility that ADAM9 may also cause conformational changes in either ACE2 or Spike proteins, thereby making it more easily accessible for virus binding. It will be interesting to investigate this possibility.

Other ADAM family members have likewise been reported to aid SARS-CoV-2 virus entry by using metalloproteinase inhibitors to diminish virus infection, consistent with our BB94 treatment (48, 49). Furthermore, ADAM17 and ADAM10 were able to cleave the Spike S2 subunit in a cell-free assay, and the silencing of ADAM10 reduced the metalloproteinase-dependent virus entry and syncytia formation. Therefore, ADAM10 is proposed to mediate a TMPRSS2-independent cell surface pathway. In contrast, our data show that instead of the fusion pathway, ADAM9 may be involved in the virus binding and endocytosis stages of SARS-CoV-2 infection. Several possibilities may explain the divergent roles of the ADAM family members: (i) compared to most of the ADAMs, the ADAM10 and ADAM17 lack the EGF-like domain, though the function of this domain is yet to be studied and (ii) different ADAMs have different substrate recognition properties (16). ADAM17 has been proven to cleave ACE2 (50, 51). In contrast, even though our data showed that the metalloprotease activity of ADAM9 is important in virus entry, our preliminary data suggest that ADAM9 does not cleave Spike or ACE2 (data not shown), with the latter already demonstrated in a previous study (51). Whether ADAM9 can cleave other substrates to facilitate SARS-CoV-2 entry is still unclear and worthy of exploration. Further studies are required to decipher the different roles of the various ADAM family members in the SARS-CoV-2 infection.

ADAM9, which is ubiquitously expressed in human tissues, is highly expressed in several types of cancer, such as lung, breast, renal, and gastric cancers (52 – 55). Numerous studies state that cancer patients have a higher risk of severe COVID-19 and a higher mortality rate than the general population (56 – 58). It is, thus, tempting to speculate that the upregulation of ADAM9 in cancer patients may aggravate SARS-CoV-2 infection-causing COVID-19 severity, which still requires further evidence.

Although we demonstrated that knockdown of ADAM9 has significantly diminished SARS-CoV-2 infection, the effect is quite modest. Perhaps knockout of ADAM9 would show a stronger effect, similar to the previous ADAM9 studies that revealed the total abolishment of EMCV infection in ADAM9 knockout cells (17, 18). Nonetheless, our discovery of ADAM9’s role in SARS-CoV-2 infection provides new insights into the understanding of virus-host interactions. Since the deletion of ADAM9 in mice has shown no abnormalities (59), our work highlights ADAM9 as a potential drug target against SARS-CoV-2 infection.

## MATERIALS AND METHODS

### Cell culture

Human lung adenocarcinoma (H1650) cells and human embryonic kidney (HEK293T) cells were maintained in RPMI and Dulbecco’s modified Eagle’s medium (DMEM) (Gibco), respectively. Both media were supplemented with 10% fetal bovine serum (FBS) and antibiotics (100 U/mL penicillin G and 100 µg/mL streptomycin). Stable ADAM9 KD H1650 and HEK293T cells were generated by transduction of lentiviruses carrying pLKO.1-shADAM9 and maintained in the presence of 3 µg/mL puromycin. H1650 cells stably expressing human ACE2 (H1650-ACE2) were generated by transduction of pseudotyped lentiviruses, which carry ACE2 RNA, and maintained in the presence of 5 µg/mL blasticidin. All the cells were cultured under 5% CO_2_ at 37°C.

### Plasmids and viruses

All of the shRNA plasmids and viruses used for the arrayed shRNA screen were provided by RNA Technology Platform and Gene Manipulation Core, Academia Sinica, Taiwan. The two pLKO.1-shRNA vectors used for knockdown of ADAM9 are the following: TRCN0000046980 (shADAM9#1) and TRCN0000290528 (shADAM9#2). The pLKO.1-shLacZ control plasmid is TRCN0000072240 (shLacZ). The construction of pLNCX-LS-HA-ADAM9 and its catalytically inactive mutant pLNCX-LS-HA-ADAM9-E348A was performed by PCR amplification and site-directed mutagenesis as described previously (60). pLAS2w.ACE2.Pbsd and pcDNA(TM)3.1(+)−2019-nCoV-S (Wuhan strain wild type, B.1.1.7, B.1.617.2, and B.1.1.529) were provided by RNA Technology Platform and Gene Manipulation Core, Academia Sinica, Taiwan. The construction of pCAG.2-SARS-2-S-Flag, pCAG.2-ACE2-Flag, pCAG.2-SARS-2-S-HA, and pCAG.2-ACE2-HA was performed by amplifying the desired sequences from pcDNA(TM)3.1(+)−2019-nCoV-S and pLAS2w.ACE2.Pbsd using PCR amplification and inserted into the NheI and PmeI sites of pCAG.2 for SARS-2-S-Flag and ACE2-Flag, NheI and EcoRI for SARS-2-S-HA, and NheI and XhoI for ACE2-HA. The construction of pLKO-AS3w-SARS-2-S1-Flag.bsd, pLKO-AS3w-SARS-2-S2-Flag.bsd, and pLKO-AS3w-LS-HA-ADAM9, which were used in the immunoprecipitation assay of Spike domains, was also performed by PCR amplification and inserted into the NheI and PmeI sites of pLKO-AS3w.bsd. The sequences of all the primers are listed in Table S2. The constructs were verified by Sanger sequencing. To generate Vpps, pcDNA3.1-S (with truncation of C-terminal 18 amino acids), pCMV-d8.9, and pLAS3w-FLuc.puro were cotransfected into HEK293T cells using TransIT-LT1 Transfection Reagent (Mirus Bio) as previously described (61).

### Antibodies and reagents

Rabbit anti-ADAM9, anti-Flag tag, and mouse anti-Flag tag antibodies were purchased from Cell Signaling Technology (2099s, 2368s, and 8146s). Mouse anti-HIV p24 antibody was purchased from Abcam (ab9071). Rabbit anti-GAPDH antibody was purchased from GeneTex (GTX100118). Rabbit anti-HA tag antibody was purchased from Millipore (04–902). Normal rabbit IgG was purchased from Santa Cruz (sc-2027). Goat anti-ACE2 antibody was purchased from R&D Systems (AF933). All Alexa Fluor-conjugated secondary antibodies used for immunofluorescence were procured from Molecular Probes (Invitrogen). NH_4_Cl, BafA1, and poly-L-lysine solution (P4707) were purchased from Sigma-Aldrich. Batimastat (BB-94) was purchased from Calbiochem (196440).

### shRNA screen

H1650 and HEK293T cells were seeded at a density of 5,000 cells per well in a 96-well assay plate, transduced with arrayed shRNA-carrying lentiviruses, and treated with 3 µg/mL puromycin. At 3 days post-selection, SARS-CoV-2 Spike Vpps (MOI 0.5–1) were spinoculated for 30 min onto the cells and then incubated at 37°C overnight. Media were exchanged for complete culture media after the overnight incubation. Parallel cultures were incubated without Vpp for the MTS assay. At 72 hpi, luciferase activity and the MTS assay were measured as described below.

### Luciferase and MTS assay

To detect luciferase activity in Vpp-infected cells, the cells were lysed for 2 min at room temperature using the Bright-Glo Luciferase Assay System (Promega). An equal amount of luciferase substrate was added to an equal amount of culture media per well. The luminescence was measured using the Synergy H4 Hybrid Microplate Reader (BioTek). MTS assay was used to quantify viable cells and normalize luminescence. The culture media per well were exchanged for MTS (Promega) reagent, (3-(4,5-dimethylthiazol-2-yl)−5-(3-carboxymethoxyphenyl)−2-(4-sulfophenyl)−2H-tetrazolium), which was diluted using DMEM without phenol red (Gibco) to a ratio of 1:9. Absorbance was determined at 490 nm using the microplate reader when the MTS turned brown. Values from control cells were calculated at 100% infectivity and viability.

### RT-qPCR

Total cellular RNA was extracted using TRIzol reagent (Ambion), followed by the addition of chloroform and centrifugation. The clear upper aqueous layer, which contains the nucleic acid, was transferred to a new tube, and glycogen and isopropanol were added to precipitate the RNA. After incubation at 4°C for at least an hour, the RNA was pelleted by centrifugation, washed twice with 75% ethanol, and resuspended in nuclease-free water. RNA concentration was measured using NanoDrop 2000c Spectrophotometer (ThermoFisher). cDNA synthesis was conducted using M-MLV Reverse Transcriptase (Invitrogen) and the primer oligo (dT)20. We followed the standard TaqMan method with the Universal Probe Library system (Roche) for quantitative PCR analysis. GAPDH was used as a normalization control for cellular mRNA. The primers and probes used are listed in Table S3.

### Western blotting

Cell lysates were extracted using M-PER Mammalian Protein Extraction Reagent (ThermoFisher) containing a 50× protease inhibitor (Roche). Then, 4× Laemmli Sample Buffer (Bio-rad) and 2-mercaptoethanol (Aldrich) were added to the cell lysates before boiling at 95°C. Proteins were then subjected to SDS-PAGE and transferred to a PVDF membrane. Five percent skimmed milk in PBST was used for blocking and dilution of antibodies. The membranes were probed with the indicated primary and secondary antibodies, and signals were detected using Immobilon Western Chemiluminescent HRP Substrate (Millipore). Images were obtained using ImageQuant LAS 4000 (GE Healthcare Life Sciences).

### Immunofluorescence staining and proximity ligation assay

Cells were fixed with 4% paraformaldehyde/PBS for 20 min, permeabilized with 0.5% Triton X-100 for 5 min, blocked with 1% BSA for 30 min, and then probed with the indicated primary antibody overnight at 4°C with constant rocking. Secondary antibodies were applied for 1 h at room temperature with an appropriate Alexa Fluor-conjugated antibody. Quantification of immunofluorescence was performed by calculating the corrected total cell fluorescence = integrated density – (area of selected cell × mean fluorescence of background readings) (62). Co-localization analysis was performed using the JACoP plugin from ImageJ using Pearson’s correlation coefficient (63). For PLA, cells were stained using Duo-link proximity ligation assay (DUO92101, Sigma-Aldrich) with primary antibodies to HA and Flag tags according to the manufacturer’s protocol. Images were captured using a confocal microscope.

### Vpp infection in plasmid-transfected cells

Ninety percent confluent H1650 cells in 12-well plates were transfected with the indicated plasmids using Lipofectamine 2000 Transfection Reagent (Invitrogen), incubated for 4 h at 37°C, and reseeded onto 96-well plates. Vpps were spinoculated onto the cells following overnight incubation. Media were then exchanged for complete culture media the next day, and luciferase activity was measured at 48 hpi.

### Binding assay

ADAM9 KD H1650-ACE2 cells were seeded onto chamber slides and incubated with Vpps for 2 h at 4°C. After incubation, the unbound virions were removed by washing the cells with PBS. The cells were then immediately fixed for immunofluorescence staining.

### Antibody competition assay

Cells were pretreated with either rabbit anti-ADAM9 antibody or rabbit IgG as a control at 37°C. After a 1 h incubation, Spike Vpp was spinoculated onto the cells for 30 min, and the cells were incubated at 37°C for 1 h. The media were subsequently exchanged with complete culture media, and the luciferase assay was determined at 72 hpi.

### Cell-cell fusion assay

HEK293T cells were transfected with pcDNA(TM)3.1(+)−2019-nCoV-S and GFP using TransIT-LT1 Transfection Reagent (Mirus Bio). At 2 days post-transfection, the transfected cells were detached by pipetting and co-cultured onto ADAM9 KD H1650-ACE2 cells for 2.5 h at 37°C. Images were taken using a fluorescence microscope (Nikon Eclipse Ti-U).

### Immunoprecipitation assay

Cell lysates were harvested using M-PER containing protease inhibitors. Debris was removed by centrifugation, and the supernatant was subjected to immunoprecipitation with Pierce Anti-HA Agarose (Roche Diagnostics) at 4°C overnight. The immunoprecipitates were washed the next day with wash buffer I [50 mM Tris (pH 7.5), 150 mM NaCl, and 0.1% Triton X-100] and analyzed by western blotting.

### Statistics

The conventional Student’s *t*-test was used to determine statistical significance. The assays are representative of data from at least three independent experiments. Data are shown as means ± standard deviations (SDs). Statistically significant values were considered when *P* < 0.05 (*), *P* < 0.01 (**), and *P* < 0.001 (***).

## AFTER EPUB

*[This article was published on 15 September 2023 with errors in reference 48. The reference was corrected in the current version, posted on 21 September 2023.]*

## SUPPLEMENTAL MATERIAL

Supplemental figures and tables spectrum.03854-22-s0001.pdf Figures S1 to S3; Tables S1 to S3.

Supplemental file spectrum.03854-22-s0002.xlsx shRNA screening results.

ASM does not own the copyrights to Supplemental Material that may be linked to, or accessed through, an article. The authors have granted ASM a non-exclusive, world-wide license to publish the Supplemental Material files. Please contact the corresponding author directly for reuse.

## References

[1] Garcia-Beltran WF , Lam EC , St Denis K , Nitido AD , Garcia ZH , Hauser BM , Feldman J , Pavlovic MN , Gregory DJ , Poznansky MC , Sigal A , Schmidt AG , Iafrate AJ , Naranbhai V , Balazs AB . 2021. Multiple SARS-CoV-2 variants escape neutralization by vaccine-induced humoral immunity. Cell 184:2372–2383. doi:10.1016/j.cell.2021.03.013 33743213 PMC7953441

[2] Andrews N , Stowe J , Kirsebom F , Toffa S , Rickeard T , Gallagher E , Gower C , Kall M , Groves N , O’Connell A-M , Simons D , Blomquist PB , Zaidi A , Nash S , Iwani Binti Abdul Aziz N , Thelwall S , Dabrera G , Myers R , Amirthalingam G , Gharbia S , Barrett JC , Elson R , Ladhani SN , Ferguson N , Zambon M , Campbell CNJ , Brown K , Hopkins S , Chand M , Ramsay M , Lopez Bernal J . 2022. Covid-19 vaccine effectiveness against the Omicron (B.1.1.529) variant. N Engl J Med 386:1532–1546. doi:10.1056/NEJMoa2119451 35249272 PMC8908811

[3] Collie S , Champion J , Moultrie H , Bekker L-G , Gray G . 2022. Effectiveness of BNT162b2 vaccine against Omicron variant in South Africa. N Engl J Med 386:494–496. doi:10.1056/NEJMc2119270 34965358 PMC8757569

[4] Pouwels KB , Pritchard E , Matthews PC , Stoesser N , Eyre DW , Vihta K-D , House T , Hay J , Bell JI , Newton JN , Farrar J , Crook D , Cook D , Rourke E , Studley R , Peto TEA , Diamond I , Walker AS . 2021. Effect of Delta variant on viral burden and vaccine effectiveness against new SARS-CoV-2 infections in the UK. Nat Med 27:2127–2135. doi:10.1038/s41591-021-01548-7 34650248 PMC8674129

[5] Shang J , Wan Y , Luo C , Ye G , Geng Q , Auerbach A , Li F . 2020. Cell entry mechanisms of SARS-CoV-2. Proc Natl Acad Sci U S A 117:11727–11734. doi:10.1073/pnas.2003138117 32376634 PMC7260975

[6] Ou X , Liu Y , Lei X , Li P , Mi D , Ren L , Guo L , Guo R , Chen T , Hu J , Xiang Z , Mu Z , Chen X , Chen J , Hu K , Jin Q , Wang J , Qian Z . 2020. Characterization of spike glycoprotein of SARS-CoV-2 on virus entry and its immune cross-reactivity with SARS-CoV. Nat Commun 11:1620. doi:10.1038/s41467-020-15562-9 32221306 PMC7100515

[7] Murgolo N , Therien AG , Howell B , Klein D , Koeplinger K , Lieberman LA , Adam GC , Flynn J , McKenna P , Swaminathan G , Hazuda DJ , Olsen DB . 2021. SARS-CoV-2 tropism, entry, replication, and propagation: considerations for drug discovery and development. PLoS Pathog 17:e1009225. doi:10.1371/journal.ppat.1009225 33596266 PMC7888651

[8] Hoffmann M , Kleine-Weber H , Pöhlmann S . 2020. A multibasic cleavage site in the spike protein of SARS-CoV-2 is essential for infection of human lung cells. Mol Cell 78:779–784. doi:10.1016/j.molcel.2020.04.022 32362314 PMC7194065

[9] Bestle D , Heindl MR , Limburg H , Van Lam van T , Pilgram O , Moulton H , Stein DA , Hardes K , Eickmann M , Dolnik O , Rohde C , Klenk H-D , Garten W , Steinmetzer T , Böttcher-Friebertshäuser E . 2020. TMPRSS2 and furin are both essential for proteolytic activation of SARS-CoV-2 in human airway cells. Life Sci Alliance 3:e202000786. doi:10.26508/lsa.202000786 32703818 PMC7383062

[10] Hoffmann M , Kleine-Weber H , Schroeder S , Krüger N , Herrler T , Erichsen S , Schiergens TS , Herrler G , Wu N-H , Nitsche A , Müller MA , Drosten C , Pöhlmann S . 2020. SARS-CoV-2 cell entry depends on ACE2 and TMPRSS2 and is blocked by a clinically proven protease inhibitor. Cell 181:271–280. doi:10.1016/j.cell.2020.02.052 32142651 PMC7102627

[11] Zhao M-M , Yang W-L , Yang F-Y , Zhang L , Huang W-J , Hou W , Fan C-F , Jin R-H , Feng Y-M , Wang Y-C , Yang J-K . 2021. Cathepsin L plays a key role in SARS-CoV-2 infection in humans and humanized mice and is a promising target for new drug development. Signal Transduct Target Ther 6:134. doi:10.1038/s41392-021-00558-8 33774649 PMC7997800

[12] Yadav R , Chaudhary JK , Jain N , Chaudhary PK , Khanra S , Dhamija P , Sharma A , Kumar A , Handu S . 2021. Role of structural and non-structural proteins and therapeutic targets of SARS-CoV-2 for COVID-19. Cells 10:821. doi:10.3390/cells10040821 33917481 PMC8067447

[13] Sungnak W , Huang N , Bécavin C , Berg M , Queen R , Litvinukova M , Talavera-López C , Maatz H , Reichart D , Sampaziotis F , Worlock KB , Yoshida M , Barnes JL , HCA Lung Biological Network . 2020. SARS-CoV-2 entry factors are highly expressed in nasal epithelial cells together with innate immune genes. Nat Med 26:681–687. doi:10.1038/s41591-020-0868-6 32327758 PMC8637938

[14] Hikmet F , Méar L , Edvinsson Å , Micke P , Uhlén M , Lindskog C . 2020. The protein expression profile of ACE2 in human tissues. Mol Syst Biol 16:e9610. doi:10.15252/msb.20209610 32715618 PMC7383091

[15] Wang Y , Wang Y , Luo W , Huang L , Xiao J , Li F , Qin S , Song X , Wu Y , Zeng Q , Jin F , Wang Y . 2020. A comprehensive investigation of the mRNA and protein level of ACE2, the putative receptor of SARS-CoV-2, in human tissues and blood cells. Int J Med Sci 17:1522–1531. doi:10.7150/ijms.46695 32669955 PMC7359402

[16] Chou C-W , Huang Y-K , Kuo T-T , Liu J-P , Sher Y-P . 2020. An overview of ADAM9: structure, activation, and regulation in human diseases. Int J Mol Sci 21:7790. doi:10.3390/ijms21207790 33096780 PMC7590139

[17] Baggen J , Thibaut HJ , Hurdiss DL , Wahedi M , Marceau CD , van Vliet ALW , Carette JE , van Kuppeveld FJM . 2019. Identification of the cell-surface protease ADAM9 as an entry factor for encephalomyocarditis virus. mBio 10:e01780-19. doi:10.1128/mBio.01780-19 31409686 PMC6692517

[18] Bazzone LE , King M , MacKay CR , Kyawe PP , Meraner P , Lindstrom D , Rojas-Quintero J , Owen CA , Wang JP , Brass AL , Kurt-Jones EA , Finberg RW . 2019. A disintegrin and metalloproteinase 9 domain (ADAM9) is a major susceptibility factor in the early stages of encephalomyocarditis virus infection. mBio 10:e02734-18. doi:10.1128/mBio.02734-18 30723129 PMC6428755

[19] Liu J , Li Y , Liu Q , Yao Q , Wang X , Zhang H , Chen R , Ren L , Min J , Deng F , Yan B , Liu L , Hu Z , Wang M , Zhou Y . 2021. SARS-CoV-2 cell tropism and multiorgan infection. Cell Discov 7:17. doi:10.1038/s41421-021-00249-2 33758165 PMC7987126

[20] Puelles VG , Lütgehetmann M , Lindenmeyer MT , Sperhake JP , Wong MN , Allweiss L , Chilla S , Heinemann A , Wanner N , Liu S , Braun F , Lu S , Pfefferle S , Schröder AS , Edler C , Gross O , Glatzel M , Wichmann D , Wiech T , Kluge S , Pueschel K , Aepfelbacher M , Huber TB . 2020. Multiorgan and renal tropism of SARS-CoV-2. N Engl J Med 383:590–592. doi:10.1056/NEJMc2011400 32402155 PMC7240771

[21] Shulla A , Heald-Sargent T , Subramanya G , Zhao J , Perlman S , Gallagher T . 2011. A transmembrane serine protease is linked to the severe acute respiratory syndrome Coronavirus receptor and activates virus entry. J Virol 85:873–882. doi:10.1128/JVI.02062-10 21068237 PMC3020023

[22] Xia S , Lan Q , Su S , Wang X , Xu W , Liu Z , Zhu Y , Wang Q , Lu L , Jiang S . 2020. The role of furin cleavage site in SARS-CoV-2 spike protein-mediated membrane fusion in the presence or absence of trypsin. Signal Transduct Target Ther 5:92. doi:10.1038/s41392-020-0184-0 32532959 PMC7289711

[23] Lonsdale J , Thomas J , Salvatore M , Phillips R , Lo E , Shad S , Hasz R , Walters G , Garcia F , Young N , Foster B , Moser M , Karasik E , Gillard B , Ramsey K , Sullivan S , Bridge J , Magazine H , Syron J , Fleming J , Siminoff L , Traino H , Mosavel M , Barker L , Jewell S , Rohrer D , Maxim D , Filkins D , Harbach P , Cortadillo E , Berghuis B , Turner L , Hudson E , Feenstra K , Sobin L , Robb J , Branton P , Korzeniewski G , Shive C , Tabor D , Qi L , Groch K , Nampally S , Buia S , Zimmerman A , Smith A , Burges R , Robinson K , Valentino K , Bradbury D , Cosentino M , Diaz-Mayoral N , Kennedy M , Engel T , Williams P , Erickson K , Ardlie K , Winckler W , Getz G , DeLuca D , MacArthur D , Kellis M , Thomson A , Young T , Gelfand E , Donovan M , Meng Y , Grant G , Mash D , Marcus Y , Basile M , Liu J , Zhu J , Tu Z , Cox NJ , Nicolae DL , Gamazon ER , Im HK , Konkashbaev A , Pritchard J , Stevens M , Flutre T , Wen X , Dermitzakis ET , Lappalainen T , Guigo R , Monlong J , Sammeth M , Koller D , Battle A , Mostafavi S , McCarthy M , Rivas M , Maller J , Rusyn I , Nobel A , Wright F , Shabalin A , Feolo M , Sharopova N , Sturcke A , Paschal J , Anderson JM , Wilder EL , Derr LK , Green ED , Struewing JP , Temple G , Volpi S , Boyer JT , Thomson EJ , Guyer MS , Ng C , Abdallah A , Colantuoni D , Insel TR , Koester SE , Little AR , Bender PK , Lehner T , Yao Y , Compton CC , Vaught JB , Sawyer S , Lockhart NC , Demchok J , Moore HF . 2013. The genotype-tissue expression (GTEx) project. Nat Genet 45:580–585. doi:10.1038/ng.2653 23715323 PMC4010069

[24] Su W-C , Chen Y-C , Tseng C-H , Hsu PW-C , Tung K-F , Jeng K-S , Lai MMC . 2013. Pooled RNAi screen identifies ubiquitin ligase Itch as crucial for influenza A virus release from the endosome during virus entry. Proc Natl Acad Sci U S A 110:17516–17521. doi:10.1073/pnas.1312374110 24101521 PMC3808593

[25] Hao L , Sakurai A , Watanabe T , Sorensen E , Nidom CA , Newton MA , Ahlquist P , Kawaoka Y . 2008. Drosophila RNAi screen identifies host genes important for influenza virus replication. Nature 454:890–893. doi:10.1038/nature07151 18615016 PMC2574945

[26] Edinger TO , Pohl MO , Yángüez E , Stertz S . 2015. Cathepsin W is required for escape of influenza A virus from late endosomes. mBio 6:e00297. doi:10.1128/mBio.00297-15 26060270 PMC4462628

[27] Krishnan MN , Ng A , Sukumaran B , Gilfoy FD , Uchil PD , Sultana H , Brass AL , Adametz R , Tsui M , Qian F , Montgomery RR , Lev S , Mason PW , Koski RA , Elledge SJ , Xavier RJ , Agaisse H , Fikrig E . 2008. RNA interference screen for human genes associated with West Nile virus infection. Nature 455:242–245. doi:10.1038/nature07207 18690214 PMC3136529

[28] König R , Stertz S , Zhou Y , Inoue A , Hoffmann H-H , Bhattacharyya S , Alamares JG , Tscherne DM , Ortigoza MB , Liang Y , Gao Q , Andrews SE , Bandyopadhyay S , De Jesus P , Tu BP , Pache L , Shih C , Orth A , Bonamy G , Miraglia L , Ideker T , García-Sastre A , Young JAT , Palese P , Shaw ML , Chanda SK . 2010. Human host factors required for influenza virus replication. Nature 463:813–817. doi:10.1038/nature08699 20027183 PMC2862546

[29] Li Q , Brass AL , Ng A , Hu Z , Xavier RJ , Liang TJ , Elledge SJ . 2009. A genome-wide genetic screen for host factors required for hepatitis C virus propagation. Proc Natl Acad Sci U S A 106:16410–16415. doi:10.1073/pnas.0907439106 19717417 PMC2752535

[30] Tai AW , Benita Y , Peng LF , Kim S-S , Sakamoto N , Xavier RJ , Chung RT . 2009. A functional genomic screen identifies cellular cofactors of hepatitis C virus replication. Cell Host Microbe 5:298–307. doi:10.1016/j.chom.2009.02.001 19286138 PMC2756022

[31] Su W-C , Hsu S-F , Lee Y-Y , Jeng K-S , Lai MMC . 2015. A nucleolar protein, ribosomal RNA processing 1 homolog B (RRP1B) enhances the recruitment of cellular mRNA in influenza virus transcription. J Virol 89:11245–11255. doi:10.1128/JVI.01487-15 26311876 PMC4645683

[32] Bohan D , Van Ert H , Ruggio N , Rogers KJ , Badreddine M , Aguilar Briseño JA , Elliff JM , Rojas Chavez RA , Gao B , Stokowy T , Christakou E , Kursula P , Micklem D , Gausdal G , Haim H , Minna J , Lorens JB , Maury W . 2021. Phosphatidylserine receptors enhance SARS-CoV-2 infection. PLoS Pathog 17:e1009743. doi:10.1371/journal.ppat.1009743 34797899 PMC8641883

[33] Mesta F , Coll AM , Ramírez MÁ , Delgado-Roche L . 2021. Predictors of mortality in hospitalized COVID-19 patients: a Mexican population-based cohort study. Biomedicine 11:1–4. doi:10.37796/2211-8039.1124 PMC8824247 35223397

[34] Carapito R , Li R , Helms J , Carapito C , Gujja S , Rolli V , Guimaraes R , Malagon-Lopez J , Spinnhirny P , Lederle A , Mohseninia R , Hirschler A , Muller L , Bastard P , Gervais A , Zhang Q , Danion F , Ruch Y , Schenck M , Collange O , Chamaraux-Tran T-N , Molitor A , Pichot A , Bernard A , Tahar O , Bibi-Triki S , Wu H , Paul N , Mayeur S , Larnicol A , Laumond G , Frappier J , Schmidt S , Hanauer A , Macquin C , Stemmelen T , Simons M , Mariette X , Hermine O , Fafi-Kremer S , Goichot B , Drenou B , Kuteifan K , Pottecher J , Mertes P-M , Kailasan S , Aman MJ , Pin E , Nilsson P , Thomas A , Viari A , Sanlaville D , Schneider F , Sibilia J , Tharaux P-L , Casanova J-L , Hansmann Y , Lidar D , Radosavljevic M , Gulcher JR , Meziani F , Moog C , Chittenden TW , Bahram S . 2022. Identification of driver genes for critical forms of COVID-19 in a deeply phenotyped young patient cohort. Sci Transl Med 14:eabj7521. doi:10.1126/scitranslmed.abj7521 34698500

[35] Kohga K , Takehara T , Tatsumi T , Ishida H , Miyagi T , Hosui A , Hayashi N . 2010. Sorafenib inhibits the shedding of major histocompatibility complex class I–related chain A on hepatocellular carcinoma cells by down-regulating a disintegrin and metalloproteinase 9. Hepatology 51:1264–1273. doi:10.1002/hep.23456 20099300

[36] Peck KM , Lauring AS . 2018. Complexities of viral mutation rates. J Virol 92:e01031-17. doi:10.1128/JVI.01031-17 29720522 PMC6026756

[37] Peng R , Wu L-A , Wang Q , Qi J , Gao GF . 2021. Cell entry by SARS-CoV-2. Trends Biochem Sci 46:848–860. doi:10.1016/j.tibs.2021.06.001 34187722 PMC8180548

[38] Wang K , Chen W , Zhang Z , Deng Y , Lian J-Q , Du P , Wei D , Zhang Y , Sun X-X , Gong L , Yang X , He L , Zhang L , Yang Z , Geng J-J , Chen R , Zhang H , Wang B , Zhu Y-M , Nan G , Jiang J-L , Li L , Wu J , Lin P , Huang W , Xie L , Zheng Z-H , Zhang K , Miao J-L , Cui H-Y , Huang M , Zhang J , Fu L , Yang X-M , Zhao Z , Sun S , Gu H , Wang Z , Wang C-F , Lu Y , Liu Y-Y , Wang Q-Y , Bian H , Zhu P , Chen Z-N . 2020. CD147-spike protein is a novel route for SARS-CoV-2 infection to host cells. Signal Transduct Target Ther 5:283. doi:10.1038/s41392-020-00426-x 33277466 PMC7714896

[39] Wang S , Qiu Z , Hou Y , Deng X , Xu W , Zheng T , Wu P , Xie S , Bian W , Zhang C , Sun Z , Liu K , Shan C , Lin A , Jiang S , Xie Y , Zhou Q , Lu L , Huang J , Li X . 2021. AXL is a candidate receptor for SARS-CoV-2 that promotes infection of pulmonary and bronchial epithelial cells. Cell Res 31:126–140. doi:10.1038/s41422-020-00460-y 33420426 PMC7791157

[40] Daly JL , Simonetti B , Klein K , Chen K-E , Williamson MK , Antón-Plágaro C , Shoemark DK , Simón-Gracia L , Bauer M , Hollandi R , Greber UF , Horvath P , Sessions RB , Helenius A , Hiscox JA , Teesalu T , Matthews DA , Davidson AD , Collins BM , Cullen PJ , Yamauchi Y . 2020. Neuropilin-1 is a host factor for SARS-CoV-2 infection. Science 370:861–865. doi:10.1126/science.abd3072 33082294 PMC7612957

[41] Cantuti-Castelvetri L , Ojha R , Pedro LD , Djannatian M , Franz J , Kuivanen S , van der Meer F , Kallio K , Kaya T , Anastasina M , Smura T , Levanov L , Szirovicza L , Tobi A , Kallio-Kokko H , Österlund P , Joensuu M , Meunier FA , Butcher SJ , Winkler MS , Mollenhauer B , Helenius A , Gokce O , Teesalu T , Hepojoki J , Vapalahti O , Stadelmann C , Balistreri G , Simons M . 2020. Neuropilin-1 facilitates SARS-CoV-2 cell entry and infectivity. Science 370:856–860. doi:10.1126/science.abd2985 33082293 PMC7857391

[42] Carlos AJ , Ha DP , Yeh D-W , Van Krieken R , Tseng C-C , Zhang P , Gill P , Machida K , Lee AS . 2021. The chaperone GRP78 is a host auxiliary factor for SARS-CoV-2 and GRP78 depleting antibody blocks viral entry and infection. J Biol Chem 296:100759. doi:10.1016/j.jbc.2021.100759 33965375 PMC8102082

[43] Yang C , Zhang Y , Zeng X , Chen H , Chen Y , Yang D , Shen Z , Wang X , Liu X , Xiong M , Chen H , Huang K . 2021. Kidney injury molecule-1 is a potential receptor for SARS-CoV-2. J Mol Cell Biol 13:185–196. doi:10.1093/jmcb/mjab003 33493263 PMC7928767

[44] Gu Y , Cao J , Zhang X , Gao H , Wang Y , Wang J , He J , Jiang X , Zhang J , Shen G , Yang J , Zheng X , Hu G , Zhu Y , Du S , Zhu Y , Zhang R , Xu J , Lan F , Qu D , Xu G , Zhao Y , Gao D , Xie Y , Luo M , Lu Z . 2022. Receptome profiling identifies KREMEN1 and ASGR1 as alternative functional receptors of SARS-CoV-2. Cell Res 32:24–37. doi:10.1038/s41422-022-00654-6 34837059 PMC8617373

[45] Mygind KJ , Schwarz J , Sahgal P , Ivaska J , Kveiborg M . 2018. Loss of ADAM9 expression impairs β1 integrin endocytosis, focal adhesion formation and cancer cell migration. J Cell Sci 131:jcs205393. doi:10.1242/jcs.205393 29142101

[46] Hirao T , Nanba D , Tanaka M , Ishiguro H , Kinugasa Y , Doki Y , Yano M , Matsuura N , Monden M , Higashiyama S . 2006. Overexpression of ADAM9 enhances growth factor-mediated recycling of E-cadherin in human colon cancer cell line HT29 cells. Exp Cell Res 312:331–339. doi:10.1016/j.yexcr.2005.10.032 16336960

[47] Mygind KJ , Störiko T , Freiberg ML , Samsøe-Petersen J , Schwarz J , Andersen OM , Kveiborg M . 2018. Sorting nexin 9 (SNX9) regulates levels of the transmembrane ADAM9 at the cell surface. J Biol Chem 293:8077–8088. doi:10.1074/jbc.RA117.001077 29622675 PMC5971451

[48] Jocher G , Grass V , Tschirner SK , Riepler L , Breimann S , Kaya T , Oelsner M , Hamad MS , Hofmann LI , Blobel CP , Schmidt-Weber CB , Gokce O , Jakwerth CA , Trimpert J , Kimpel J , Pichlmair A , Lichtenthaler SF . 2022. ADAM10 and ADAM17 promote SARS-CoV-2 cell entry and spike protein-mediated lung cell fusion. EMBO Rep 23:e54305. doi:10.15252/embr.202154305 35527514 PMC9171409

[49] Yamamoto M , Gohda J , Kobayashi A , Tomita K , Hirayama Y , Koshikawa N , Seiki M , Semba K , Akiyama T , Kawaguchi Y , Inoue J-I . 2022. Metalloproteinase-dependent and TMPRSS2-independent cell surface entry pathway of SARS-CoV-2 requires the furin cleavage site and the S2 domain of spike protein. mBio 13:e0051922. doi:10.1128/mbio.00519-22 35708281 PMC9426510

[50] Heurich A , Hofmann-Winkler H , Gierer S , Liepold T , Jahn O , Pöhlmann S . 2014. TMPRSS2 and ADAM17 cleave ACE2 differentially and only proteolysis by TMPRSS2 augments entry driven by the severe acute respiratory syndrome coronavirus spike protein. J Virol 88:1293–1307. doi:10.1128/JVI.02202-13 24227843 PMC3911672

[51] Lambert DW , Yarski M , Warner FJ , Thornhill P , Parkin ET , Smith AI , Hooper NM , Turner AJ . 2005. Tumor necrosis factor-alpha convertase (ADAM17) mediates regulated ectodomain shedding of the severe-acute respiratory syndrome-Coronavirus (SARS-CoV) receptor, angiotensin-converting enzyme-2 (ACE2)*. J Biol Chem 280:30113–30119. doi:10.1074/jbc.M505111200 15983030 PMC8062222

[52] Wang J , Zhou Y , Fei X , Chen X , Yan J , Liu B , Zhu Z . 2017. ADAM9 functions as a promoter of gastric cancer growth which is negatively and post-transcriptionally regulated by miR-126. Oncol Rep 37:2033–2040. doi:10.3892/or.2017.5460 28260063

[53] Fritzsche FR , Wassermann K , Jung M , Tölle A , Kristiansen I , Lein M , Johannsen M , Dietel M , Jung K , Kristiansen G . 2008. ADAM9 is highly expressed in renal cell cancer and is associated with tumour progression. BMC Cancer 8:179. doi:10.1186/1471-2407-8-179 18582378 PMC2442841

[54] Zhou R , Cho WCS , Ma V , Cheuk W , So Y-K , Wong SCC , Zhang M , Li C , Sun Y , Zhang H , Chan LWC , Tian M . 2020. ADAM9 mediates triple-negative breast cancer progression via AKT/NF-κB pathway. Front Med 7. doi:10.3389/fmed.2020.00214 PMC7317048 32637415

[55] Zhang J , Qi J , Chen N , Fu W , Zhou B , He A . 2013. High expression of a disintegrin and metalloproteinase-9 predicts a shortened survival time in completely resected stage I non-small cell lung cancer. Oncol Lett 5:1461–1466. doi:10.3892/ol.2013.1209 23761811 PMC3678878

[56] Mousavi SA , Rostami T , Kiumarsi A , Rad S , Rostami M , Motamedi F , Gandomi-Mohammadabadi A , Mirhosseini A . 2021. COVID-19 and cancer: a comparative case series. Cancer Treat Res Commun 27:100339. doi:10.1016/j.ctarc.2021.100339 33618150 PMC7885684

[57] Han S , Zhuang Q , Chiang J , Tan SH , Chua GWY , Xie C , Chua MLK , Soon YY , Yang VS . 2022. Impact of cancer diagnoses on the outcomes of patients with COVID-19: a systematic review and meta-analysis. BMJ Open 12:e044661. doi:10.1136/bmjopen-2020-044661 PMC8822543 35131810

[58] Dai M , Liu D , Liu M , Zhou F , Li G , Chen Z , Zhang Z , You H , Wu M , Zheng Q , Xiong Y , Xiong H , Wang C , Chen C , Xiong F , Zhang Y , Peng Y , Ge S , Zhen B , Yu T , Wang L , Wang H , Liu Y , Chen Y , Mei J , Gao X , Li Z , Gan L , He C , Li Z , Shi Y , Qi Y , Yang J , Tenen DG , Chai L , Mucci LA , Santillana M , Cai H . 2020. Patients with cancer appear more vulnerable to SARS-CoV-2: a multicenter study during the COVID-19 outbreak. Cancer Discov 10:783–791. doi:10.1158/2159-8290.CD-20-0422 32345594 PMC7309152

[59] Weskamp G , Cai H , Brodie TA , Higashyama S , Manova K , Ludwig T , Blobel CP . 2002. Mice lacking the metalloprotease-disintegrin MDC9 (ADAM9) have no evident major abnormalities during development or adult life. Mol Cell Biol 22:1537–1544. doi:10.1128/MCB.22.5.1537-1544.2002 11839819 PMC134708

[60] Lin C-Y , Cho C-F , Bai S-T , Liu J-P , Kuo T-T , Wang L-J , Lin Y-S , Lin C-C , Lai L-C , Lu T-P , Hsieh C-Y , Chu C-N , Cheng D-C , Sher Y-P . 2017. ADAM9 promotes lung cancer progression through vascular remodeling by VEGFA, ANGPT2, and PLAT. Sci Rep 7:15108. doi:10.1038/s41598-017-15159-1 29118335 PMC5678093

[61] Melano I , Kuo L-L , Lo Y-C , Sung P-W , Tien N , Su W-C . 2021. Effects of basic amino acids and their derivatives on SARS-CoV-2 and influenza-A virus infection. Viruses 13:1301. doi:10.3390/v13071301 34372507 PMC8310019

[62] McCloy RA , Rogers S , Caldon CE , Lorca T , Castro A , Burgess A . 2014. Partial inhibition of Cdk1 in G2 phase overrides the SAC and decouples mitotic events. Cell Cycle 13:1400–1412. doi:10.4161/cc.28401 24626186 PMC4050138

[63] Bolte S , Cordelières FP . 2006. A guided tour into subcellular colocalization analysis in light microscopy. J Microsc 224:213–232. doi:10.1111/j.1365-2818.2006.01706.x 17210054

