# Supplementary material for: A disintegrin and metalloproteinase domain 9 facilitates SARS-CoV-2 entry into cells with low ACE2 expression
Source: Microbiol Spectr. 2023 Sep 15;11(5):e03854-22. doi: 10.1128/spectrum.03854-22 (PMC10581035; doi:10.1128/spectrum.03854-22)
Supplement: Supplemental figures and tables — Figures S1 to S3; Tables S1 to S3. [file spectrum.03854-22-s0001.pdf]

Figure S1

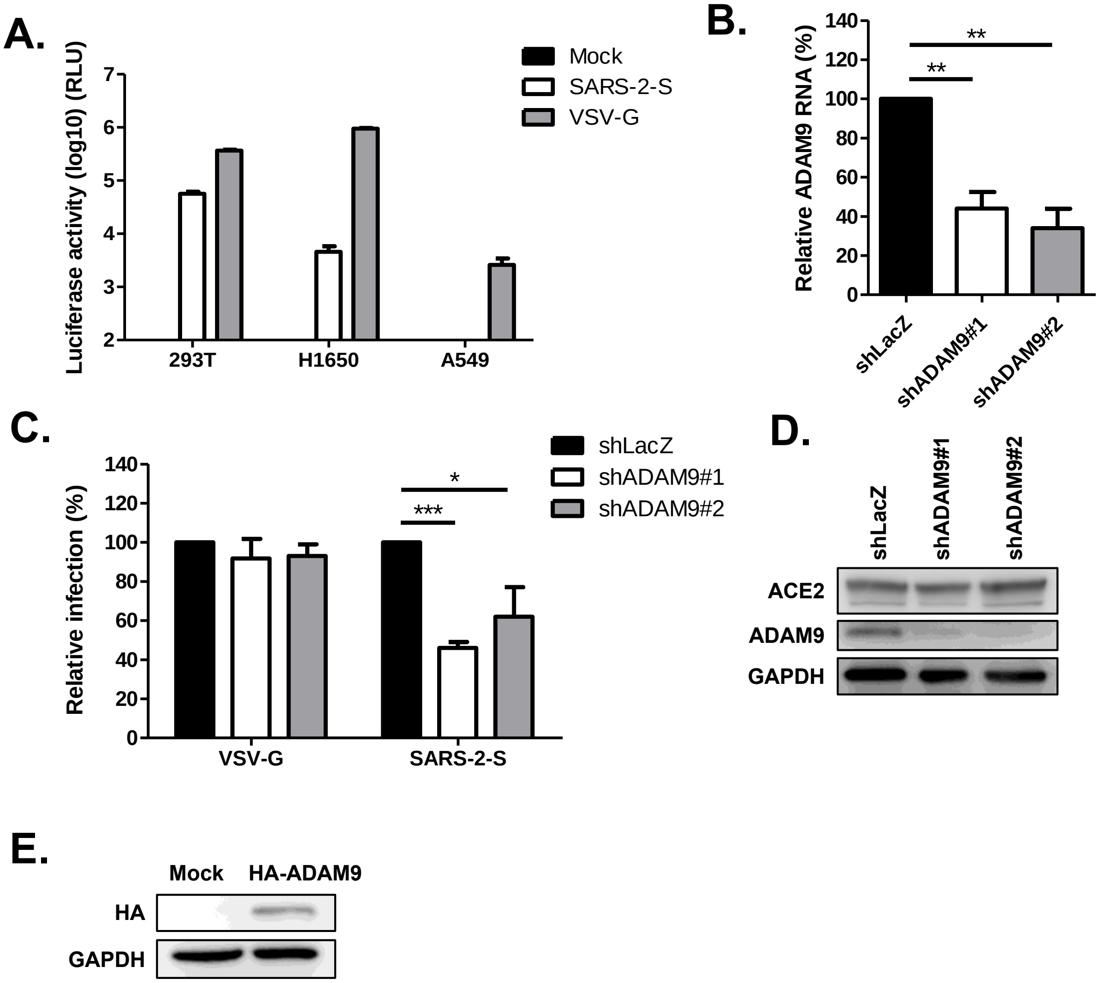

FIG S1 (A) Susceptibility of HEK293T, H1650 and A549 cells to virus pseudotype (Vpp) infection. The cells were infected with mock, SARS-CoV-2 Spike, and VSV-G Vpps. Luciferase accumulations were evaluated at 72 h post-infection (hpi). (n=3) (B) RNA of shRNA-transduced HEK293T cells were extracted after puromycin selection and ADAM9 expression levels were analyzed by RT-qPCR. The level of ADAM9 RNA was normalized by GAPDH RNA. (n=2) (C) shLacZ control and ADAM9 knockdown HEK293T cells were transduced with Spike or VSV-G Vpps, and luciferase accumulations were evaluated at 48 hpi. Each infection level was compared with that of the control, shLacZ. Values represent the mean ± SD of three independent experiments. **, $P<0.01$;

and ***, P<0.001 compared with controls (n=3). (D) Cellular lysates of stable ADAM9 KD H1650-ACE2 cells were extracted after puromycin selection and ADAM9 and ACE2 protein expression levels were analyzed by western blotting (in relation to Fig. 1E and 2A). (E) Expression level of HA-ADAM9 in the plasmid-transfected H1650 cells (in relation to Fig. 1F) (D and E) GAPDH was used as the protein loading control.

Figure S2

A.

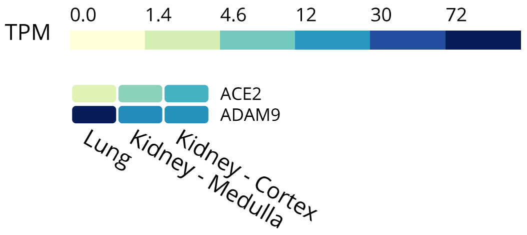

B.

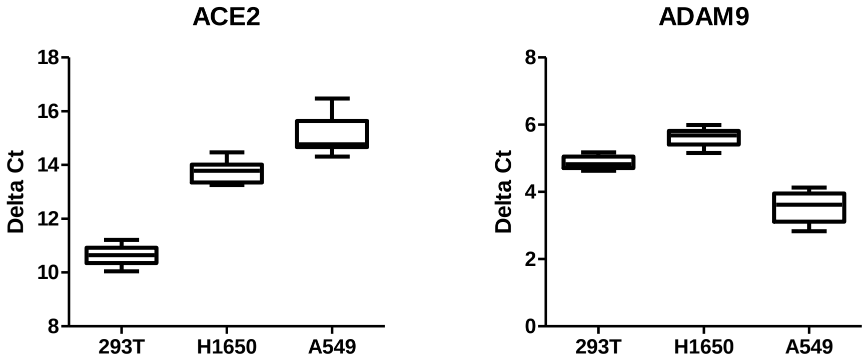

FIG S2 (A) Tissue transcriptional expression of ACE2 and ADAM9 from the Genotype-Tissue Expression (GTEx) Project. TPM, transcripts per million. (B) RNA expression of ACE2 and ADAM9 in HEK293T, H1650, and A549 cells. Values represent the mean ± SD of four independent experiments.

Figure S3

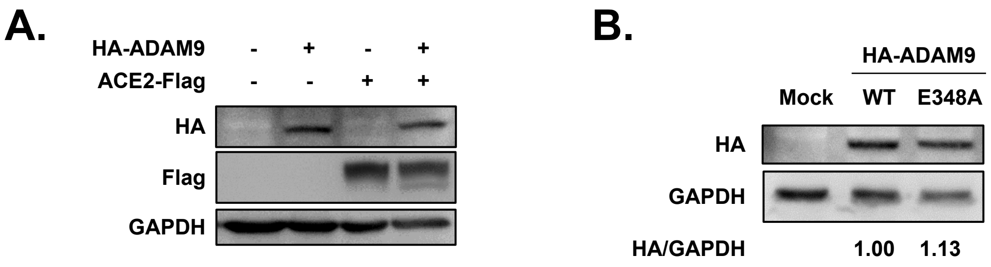

FIG S3 (A) Expression levels of HA-ADAM9 and ACE2-Flag in the plasmid-transfected H1650 cells (in relation to Fig. 4A). (B) Expression levels of HA-ADAM9-WT and HA-ADAM9-E348A in H1650 cells (in relation to Fig. 5C).

Table S1. List of top ten hits in the screens.

H1650		293T	
**Gene Symbol**	**Relative infection***	**Gene Symbol**	**Relative infection***
BACE1	7.96%	ACE2	14.85%
ACE2	8.82%	SORL1	20.57%
CTSL1	9.70%	CTSB	21.92%
ANXA2	11.78%	WNT3A	24.39%
WNT3A	14.30%	MME	26.39%
ADAM9	21.33%	PCSK9	26.77%
CAPN2	22.47%	CTSL1	27.19%
BST2	24.24%	ADAM9	27.39%
TMPRSS2	24.59%	ANXA2	31.86%
CTSB	25.62%	PCSK9	37.05%

*Average of SARS-2-Spike pseudotyped virus infection compared to shLacZ from two independent rounds of screening in the indicated cells.

Table S2. List of primers used for plasmid construction.

Primer name	Sequence (5' to 3')
**S F'**	ACTGGCTAGCGCCACCATGTTCGTCTTCCTGGTCCTGCTG
**S-Flag R'**	ACTGGTTTAAACCTACTTGTCGTCATCGTCTTTGTAGTCGGTGTAATGCAGCTTCACGC
**S-HA R'**	ACTGGAATTCGCTCCGGGCTCTTCTGGGAGAGTTTG
**S1 R'**	ACTGGTTTAAACTTACTTGTCGTCATCGTCTTTGTAGTCCCGGGCTCTTCTGGGAGAGTTTG
**S2 F'**	TGTTGCTAGCCGCCACCATGAGCGTGGCCTCCCAGTCTATC
**S2 R'**	ACTGGTTTAAACTTACTTGTCGTCATCGTCTTTGTAGTCGGTGTAATGCAGCTTCACGCC
**ACE2 F'**	ACTGGCTAGCCACCATGTCAAGCTCTTCCTGGCTCCTTC
**ACE2-HA R'**	ACTGCTCGAGCGAAAGGAGGTCTGAACATCATCAGTG
**ACE2-Flag R'**	ACTGGTTTAAACCTACTTGTCGTCATCGTCTTTGTAGTCAAAGGAGGTCTGAACATCAT
**ADAM9 F'**	ACTGGCTAGCCGCCACCATGGAGACAGACACACTCCTGC
**ADAM9 R'**	CATCGTTTAAACTCAAGTGAGGGAACTATATAAAGGAGG

Table S3. List of primers for qPCR.

Gene	Sequence (5' to 3')		Universal probe no.
**ADAM9**	Forward	TGGACCAATGGAAACCTGAT	9
	Reverse	GCCACTGCACGAAGTTCC	
**GAPDH**	Forward	AGCCACATCGCTCAGACAC	60
	Reverse	GCCCAATACGACCAAATCC	
**ACE2**	Forward	TGGGAGATGAAGCGAGAGAT	77
	Reverse	ATGCGGGGTCACAGTATGTT	
